# Transcriptomic analysis of *Staphylococcus equorum* KM1031 from the high-salt fermented seafood *jeotgal* under chloramphenicol, erythromycin and lincomycin stresses

**DOI:** 10.1038/s41598-022-19897-9

**Published:** 2022-09-15

**Authors:** Sojeong Heo, Tao Kim, Hong-Eun Na, Gawon Lee, Jong-Hoon Lee, Do-Won Jeong

**Affiliations:** 1 Department of Food and Nutrition, Dongduk Women’s University, Seoul, 02748 Republic of Korea; 2 Department of Food Science and Biotechnology, Kyonggi University, Suwon, 16227 Republic of Korea

**Keywords:** Applied microbiology, Microbial ecology

## Abstract

*Staphylococcus equorum* strain KM1031 is resistant to chloramphenicol, erythromycin and lincomycin. To shed light on the genetic factors underlying these antibiotic resistances, we determined the global gene expression profile of *S. equorum* KM1031 using RNA sequencing. During chloramphenicol, erythromycin and lincomycin treatment, 8.3% (183/2,336), 16.0% (354/2,336), and 2.9% (63/2,336) of *S. equorum* KM1031 genes exhibited significant differences in expression, respectively. These three antibiotics upregulated genes related to efflux and downregulated genes related to transporters. Antibiotic treatment also upregulated osmoprotectant-related genes involved in salt tolerance. To identify specific genes functionally related to antibiotic resistance, we compared the genome of strain KM1031 with those of three *S. equorum* strains that are sensitive to these three antibiotics. We identified three genes of particular interest: an antibiotic biosynthesis monooxygenase gene (*abm*, AWC34_RS01805) related to chloramphenicol resistance, an antibiotic ABC transporter ATP-binding protein gene (*msr*, AWC34_RS11115) related to erythromycin resistance, and a lincosamide nucleotydyltransferase gene (*lnuA*, AWC34_RS13300) related to lincomycin resistance. These genes were upregulated in response to the corresponding antibiotic; in particular, *msr* was upregulated more than fourfold by erythromycin treatment. Finally, the results of RNA sequencing were validated by quantitative real-time PCR. This transcriptomic analysis provides genetic evidence regarding antibiotic stress responses of *S. equorum* strain KM1031.

## Introduction

Four coagulase-negative staphylococci (*Staphylococcus carnosus*, *S. equorum*, *S. succinus*, and *S. xylosus*) are frequently detected in naturally fermented meat products and cheese^1–3^. These species are recognized as benign bacteria^4^. *S. equorum* is commonly used in starter cultures for meat and cheese fermentation^5,6^, and has been reported to contribute to the aromas of fermented foods through production of low-molecular-weight aromatic compounds such as esters, amino acids, aldehydes, and free fatty acids^7,8^.

*Staphylococcus equorum* has also been identified as the dominant species in *jeotgal*, a high-salt-fermented seafood produced in Korea^9^. *S. equorum* strain KS1039 was selected as a starter candidate among many *jeotgal*-derived *S. equorum* strains after a series of safety assessments^10^. These safety assessments showed that most *S. equorum* isolates from *jeotgal* were susceptible to 15 types of antibiotic and were nonhemolytic^10^. Sequencing of the complete genome of *S. equorum* strain KS1039 demonstrated the absence of virulence genes found in the well-known pathogen *S. aureus*^11^. In addition, genomic insights into strain KS1039 suggested its usefulness as a starter culture for aroma enhancement, bacteriocin production, foreign plasmid restriction, and nutrient optimization^12^.

In our previous study, we undertook a comparative genomic analysis of six phenotypically different *S. equorum* strains from Korean high-salt seafoods, including the multi-drug resistant strain KM1031, to assess the safety of *S. equorum*. Our results suggested that antibiotic resistance was linked to acquired antibiotic resistance genes^13^. However, information from genome sequencing is insufficient to capture the dynamics of gene expression under antibiotic stress. Strain KM1031 exhibited resistance to chloramphenicol, erythromycin, lincomycin and penicillin G based on disk diffusion analysis^13^. Herein, to better understand the responses of *S. equorum* strain KM1031 to these antibiotics, we conducted transcriptomic analysis of this strain following administration of each antibiotic. The results revealed expression of genes generally associated with antibiotic treatment as well as changes in the expression of specific genes depending on the antibiotic. This comparative transcriptomic study provides new insights into the antibiotic resistance mechanisms of *S. equorum*.

## Materials and methods

### Bacterial strain and culture conditions

*Staphylococcus equorum* strain KM1031 was previously isolated from the fermented seafood *myeolchi-jeotgal* and showed resistance to chloramphenicol, erythromycin, and lincomycin^10^. The complete genome sequence of strain KM1031 has been determined (GenBank accession nos. CP013980–CP013983)^13^. In this study, strain KM1031 was cultured in tryptic soy broth (TSB; BD, NJ, USA) at 30 °C for 24 h.

### Extraction and purification of RNA from *S. equorum* KM1031

An overnight culture of *S. equorum* KM1031 grown in TSB was used to inoculate fresh TSB medium to a final concentration of 1% (w/v), followed by incubation at 30 °C. When the optical density at 600 nm (OD_600_) reached 0.5, the culture was divided in three and chloramphenicol (15 μg/mL), erythromycin (5 μg/mL), and lincomycin (30 μg/mL) were added to each tube. Thereafter, the cells were further incubated at 30 °C for 2 h. Controls were prepared using the same conditions without antibiotics. Cells were collected by centrifugation and total RNA was extracted using TRIzol™ reagent (Invitrogen, Carlsbad, CA, USA) after treatment with lysostaphin (40 μg/mL) at 37 °C for 20 min according to the manufacturer’s instructions.

Total RNA from each sample was subjected to an rRNA-removal process based on the subtractive hybridization/bead capture system of the Ribo-Zero kit (Epicentre Biotechnologies, Madison, WI, USA). Purified RNA samples were used for mRNA-Seq library construction using the Illumina TruSeq RNA Sample Preparation kit v2 (Illumina, San Diego, CA, USA). RNA-Seq was performed using two Illumina HiSeq runs to generate single-end reads around 100 bp in length. All RNA-Seq data analyzed in this study, including whole transcriptome profiles (Supplementary Tables S1 and S2), were deposited in the Sequence Read Archive (SRR10807062–SRR10807065). Using the CLRNASeq program (ChunLab, Seoul, South Korea), sequencing reads were mapped to the *S. equorum* KM1031 genome and normalized. The normalization methods used in the RNA-Seq analysis included Reads Per Kilobase of transcript per Million mapped reads (RPKM), Relative Log Expression (RLE), and Trimmed Mean of *M*-value (TMM) (Table S3). Because the coefficient of variation values for the RLE and TMM methods were lower than that for RPKM and because TMM was previously reported to be the most effective normalization method^14^, TMM was used for normalization of gene expression levels. The *p*-value for TMM was calculated using edgeR and the fold-change value was calculated as [TMM_antibiotic_/TMM_control_]. For subsequent experiments, differentially expressed genes (DEGs) with an absolute log_2_ [fold change] > 2 were filtered and visualized using the CLRNASeq program. Clusters of orthologous groups (COG) analysis^15^ was used for functional grouping of all strain KM1031 genes. The proportion of DEGs in each functional group was calculated.

### Quantitative real-time PCR (qRT-PCR)

The expression levels of specific genes were validated using qRT-PCR. qRT-PCR was performed using a C1000 Thermal Cycler (Bio-Rad, Hercules, CA, USA) with IQ™ SYBR®Green Supermix (Bio-Rad). Thermal cycling consisted of 3 min at 95 °C, followed by 40 cycles of 10 s at 95 °C and 30 s at 60 °C. The primers used for the detection of target genes are listed in Supplementary Table S4. Expression levels of all genes were quantified in duplicate using three independent experiments. These analyses were performed on the same batches of RNA as those used for transcriptomic experiments. The 16S rRNA gene was used as the reference gene for normalization. Results were normalized using the comparative cycle threshold method^16^.

### Comparative genomics of *S. equorum* strains

For comparative genomic analysis within *S. equorum* strains, genome sequence data for strains KM1031 [chloramphenicol, erythromycin and lincomycin resistant (C^R^E^R^L^R^); GenBank accession: CP013980–CP013983], C2014 [chloramphenicol, erythromycin and lincomycin sensitive (C^S^E^S^L^S^); GenBank accession: CP013714–CP013719], and KS1039 (C^S^E^S^L^S^; GenBank accession: CP013114.1) were obtained from the NCBI database (http://ncbi.nlm.nih.gov/genomes).

### Cloning of the *abm* and *msr* genes and assessment of their roles in antibiotic resistance

To assess whether antibiotic resistance was dependent on the *abm* and *msr* genes, both full-length genes from *S. equorum* strain KM1031 were cloned and recombinantly expressed in *Escherichia coli*. PCR amplifications from strain KM1031 using specific primer sets (Supplementary Table S5) were performed using a T-3000 thermocycler (Biometra, Göttingen, Germany). The PCR mixture was prepared according to the manual for Inclone *Taq* DNA polymerase (Inclone Biotech, Daejeon, South Korea). Samples were preheated for 5 min at 95 °C and then amplified using 30 cycles of 1 min at 95 °C, 30 s at 55 °C, and 1 min at 72 °C. The PCR products for *abm* and *msr* were digested with *Xho*I and *EcoR*I, respectively, and then inserted into pYJ335 and pCL55 digested with the same enzymes. The resulting plasmids were designated pYJ335-abm and pCL55-msr, respectively.

To assess the roles of *abm* and *msr in* chloramphenicol and erythromycin resistance, respectively, *E. coli* DH5α (pYJ335-abm) and *E. coli* DH5α (pCL55-msr) cultures in Luria–Bertani medium (LB; BD) were normalized to an OD_600_ of 0.5 and diluted tenfold. A 10 μL aliquot was subcultured into LB medium containing 50 mg/L chloramphenicol or 100 mg/L erythromycin. The antibiotic resistance of *E. coli* transformants was determined by examining their growth. *E. coli* cells transformed with empty plasmids were used as negative controls.

## Results

### Comprehensive transcriptome analysis of *S. equorum* strain KM1031 under antibiotic stress

**Figure 1:**
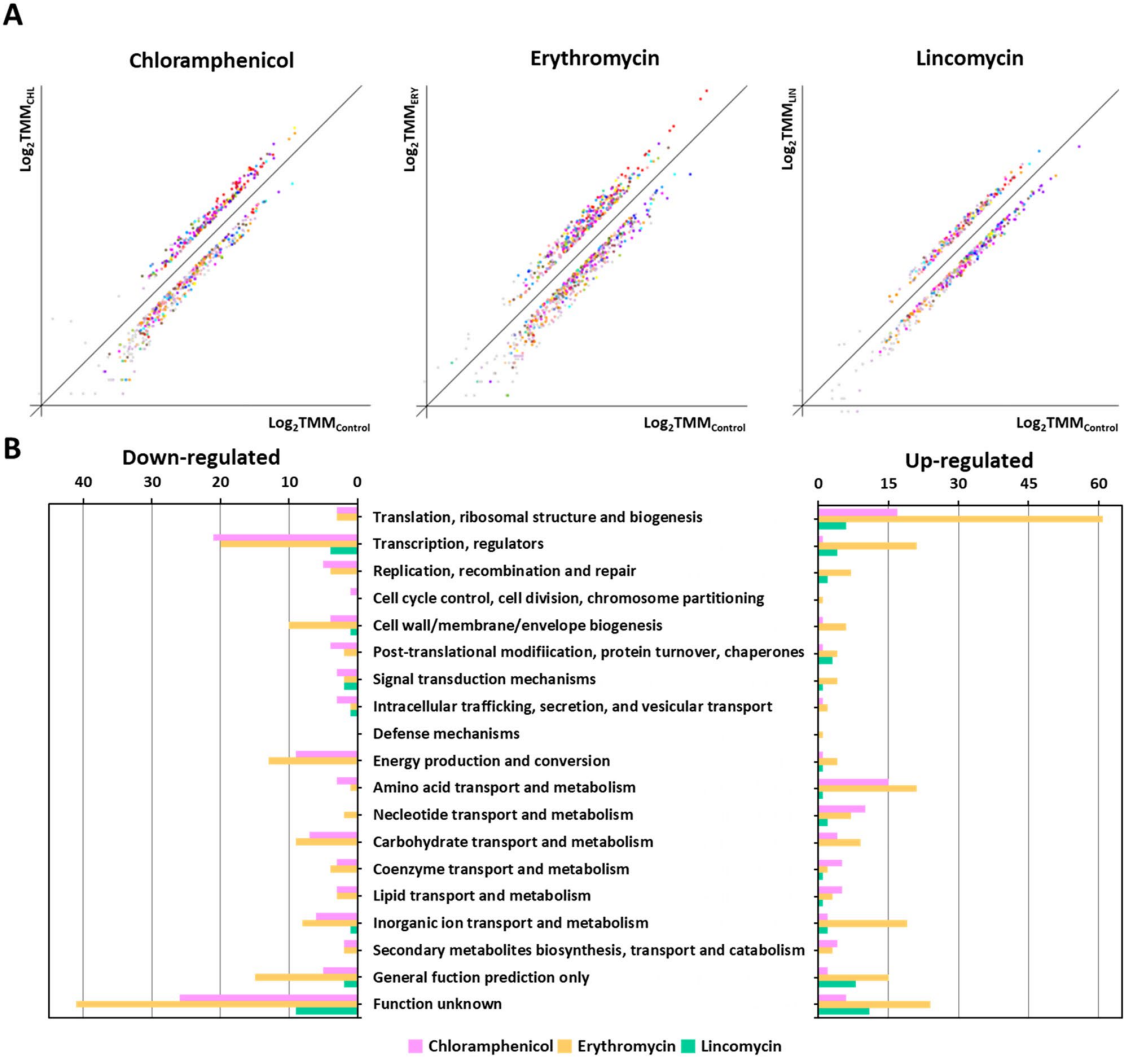
Classification of differentially expressed genes (DEGs) based on predicted functions. (**A**) DEG analysis from RNA-Seq data comparing untreated *Staphylococcus equorum* strain KM1031 with strain KM1031 treated with antibiotics. The *x*-axis shows log-scaled Trimmed Mean of *M*-value (TMM) data for strain KM1031, and the *y*-axis shows log-scaled TMM values for cells treated with chloramphenicol, erythromycin, and lincomycin, respectively. Total gene expression in the two conditions was filtered to identify significantly down- or upregulated genes with the criteria P-value ≤ 0.05 and fold-change ≥ 2. (**B**) Genes upregulated or downregulated by twofold or more following treatment of the bacterium with antibiotics were grouped into functional categories based on the Clusters of Orthologous Groups database.

In a previous study, *S. equorum* strain KM1031 showed resistance to chloramphenicol, erythromycin, lincomycin, and penicillin G based on disk diffusion analysis ^10,13^. However, in the MIC results, it was determined that strain KM1031 was sensitive to the penicillin G because it did not grow at 1 mg/L of penicillin G. To understand the bacterial response and adaptations during three antibiotic stress excluding penicillin G, RNA was isolated from *S. equorum* strain KM1031 following antibiotic stress for RNA-Seq analysis. RNA-Seq data were acquired, mapped, and normalized as described in the “Methods” section (Supplementary Tables S1 and S2). A total of 2,336 strain KM1031 genes were categorized using COG analysis. Antibiotic treatment affected the expression of several genes in strain KM1031 (Supplementary Figs. S1 and S2). After mRNA abundance was compared between control and antibiotic-exposed cells, genes showing a log_2_ (fold-change) greater than 2 or less than − 2 were considered to be DEGs (Supplementary Tables S6 and S7; Supplementary Fig. S1). In strain KM1031 cells exposed to chloramphenicol, erythromycin, and lincomycin stress, 8.3% (183/2,336), 16.0% (354/2,336), and 2.9% (63/2,336) of genes exhibited significant differences in their expression, respectively (Fig. 1B).

Following chloramphenicol treatment, 75 genes were significantly upregulated and 108 genes were significantly downregulated (Supplementary Tables S6 and S7; and Fig. 1). Significant upregulation was observed for genes associated with translation, ribosomal structure, and biogenesis (22.7%; 17/75) as well as genes associated with amino acid transport and metabolism (20.0%; 15/75). By contrast, genes associated with “function unknown” (24.1%; 26/108) and transcription (19.4%; 21/108) were downregulated.

Following erythromycin treatment, 214 genes were significantly upregulated and 140 genes were significantly downregulated. Significant upregulation was observed for genes associated with translation, ribosomal structure, and biogenesis (28.5%; 61/214) as well as genes associated with “function unknown” (11.2%; 24/214). By contrast, genes associated with “function unknown” (29.3%; 41/140) and transcription (14.3%; 20/140) were downregulated.

Following lincomycin treatment, 43 genes were significantly upregulated and 20 genes were significantly downregulated. Significant upregulation was observed for genes associated with “function unknown” (25.6%; 11/43) as well as genes associated with translation, ribosomal structure, and biogenesis (14.0%; 6/43). By contrast, genes associated with “function unknown” (45.0%; 9/20) and transcription (20.0%; 9/20) were downregulated.

### Effects of antibiotics on efflux proteins and transporters

**Figure 2:**
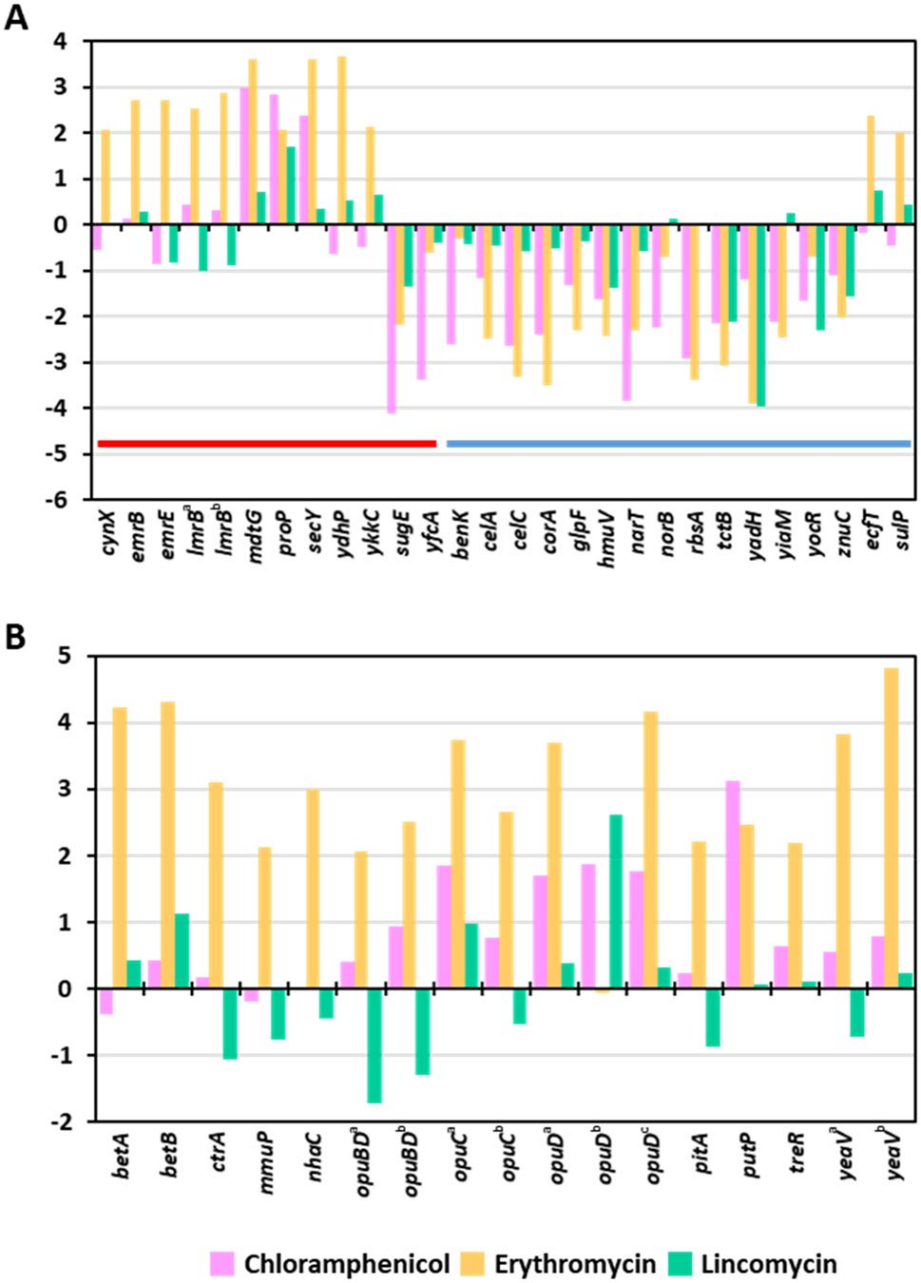
Log_2_ fold-change values for genes related to (**A**) efflux and transporters, and (**B**) salt tolerance, on treatment of *S. equorum* strain KM1031 with chloramphenicol, erythromycin and lincomycin, respectively. Color code: red: efflux-related genes, blue, transporter-related genes.

Transporter and efflux proteins are required for antibiotics to enter or be expelled from bacteria^17,18^. Thus, we hypothesized that antibiotics would alter the expression of efflux- and transporter-related genes in *S. equorum*. DEGs were screened using the keywords “efflux” and “transporter.” Among DEGs following treatment with chloramphenicol, erythromycin and lincomycin, 7.1% (13/183), 6.8% (24/354), and 4.8% (3/63), respectively, were related to transporters and efflux (Fig. 2A; Supplementary Table S8). Chloramphenicol and erythromycin treatment (especially the former) upregulated efflux-related genes and downregulated transporter-related genes. Similar results were observed for lincomycin, although expression changes were less dramatic compared with the other two antibiotics.

### Effects of antibiotics on expression of genes related to salt tolerance

Accumulation or release of compatible solutes such as glycine betaine, proline betaine, and carnitine confers salt tolerance by facilitating the response of cells to osmotic pressure^19^. Interestingly, osmoprotectant-related genes, such as those involved in the synthesis of trehalose, glycine betaine, choline, and proline, were upregulated following chloramphenicol and erythromycin treatment (Fig. 2B; Supplementary Table S8), while lincomycin only slightly affected the expression of a few genes related to salt tolerance. Zhu and Dai^20^ reported that overexpression of efflux pumps required for salt tolerance led to decreased antibiotic susceptibility. Our results suggest that strain KM1031 express salt tolerance-related genes to counter the effects of antibiotics, especially chloramphenicol and erythromycin.

### Responsive genes to three antibiotics based on transcriptomic and comparative genomic analyses

We hypothesized that some genes in *S. equorum* strain KM031 might be specifically and functionally (i.e., mechanistically) related to chloramphenicol and erythromycin resistance. To identify such genes, we undertook comparative genomic analysis of strains KM1031 (C^R^E^R^L^R^), C2014 (C^S^E^S^L^S^), and KS1039 (C^S^E^S^L^S^).

**Figure 3:**
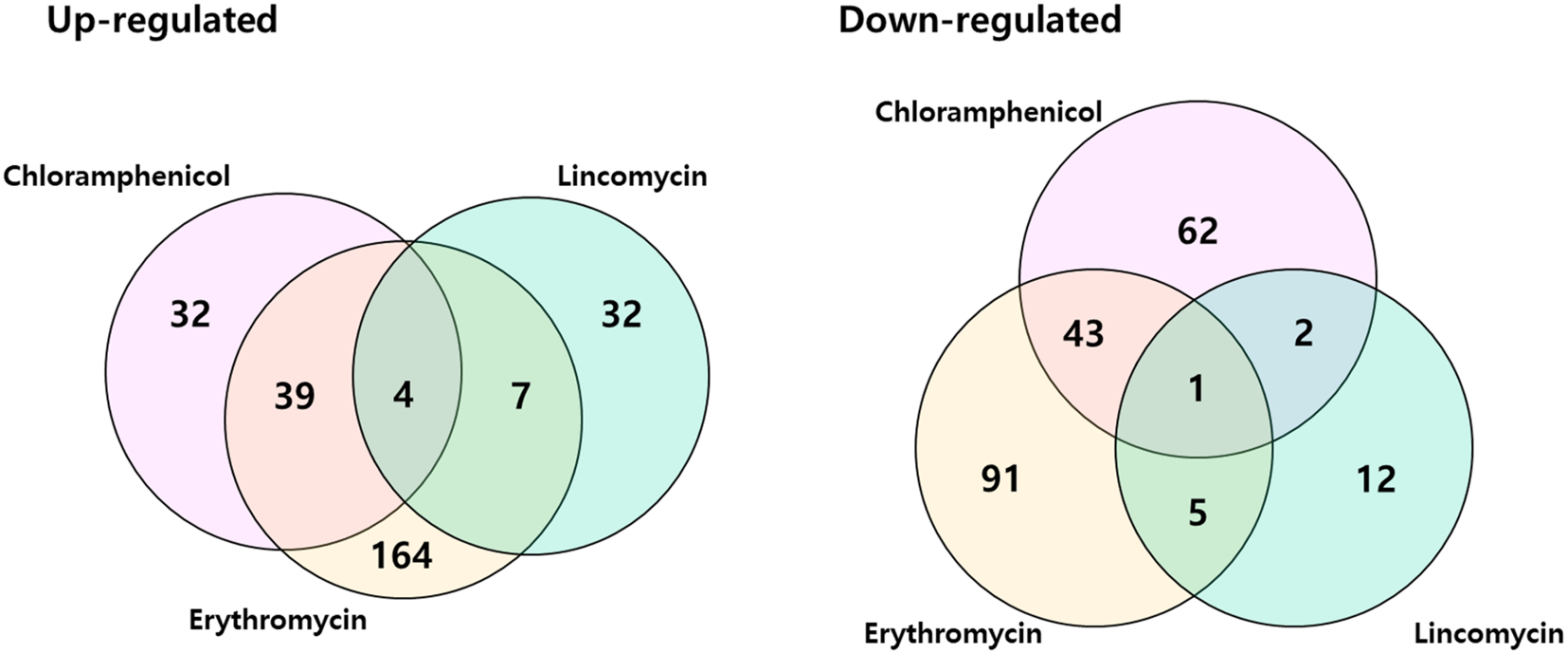
Venn diagram of differentially expressed genes (DEGs) of *S. equorum* strain KM1031 following treatment with chloramphenicol, erythromycin and lincomycin. Overlapping regions represent genes that were differentially expressed in strain KM1031 (compared with untreated cells) on treatment with two or three of the antibiotics. The numbers outside overlapping regions indicate the numbers of significantly differentially expressed genes that were affected by each antibiotic individually.

We plotted Venn diagrams of genes that were significantly differentially expressed in *S. equorum* strain KM031 in response to chloramphenicol, erythromycin and lincomycin (Fig. 3). Four genes (AWC34_RS06585, AWC34_RS08650, AWC34_RS10220, and AWC34_RS12080) were upregulated by all three antibiotics, while one (AWC34_RS11270) was downregulated by all three antibiotics (Supplementary Tables S6 and S7). These genes were detected in one or more of the complete genome sequences of the antibiotic-sensitive *S. equorum* strains C2014 and KS1039 based on comparative genomic analysis.

Interestingly, an antibiotic ABC transporter ATP-binding protein-encoding gene (*msr*, AWC34_RS11115) was identified among genes specifically upregulated in response to erythromycin. Msr is annotated, among other things, as an erythromycin resistance ATP-binding protein. This gene was suggested to be responsible for erythromycin resistance in a previous genomic study of *S. equorum* strain KM1031^13^. Reynolds et al. reported that Msr gives rise to erythromycin resistance via an active transport process^21^. Collectively, these findings strongly suggest that the antibiotic ABC transporter ATP-binding protein-coding gene (AWC34_RS11115) confers erythromycin resistance in strain KM1031. Chloramphenicol- and lincomycin-specific response genes were not identified among DEGs. Therefore, we conclude that most commonly up- and downregulated genes under antibiotic pressure are associated with general environmental responses, and not responses to chloramphenicol, erythromycin, and/or lincomycin specifically.

We hypothesized that antibiotic exposure might increase the expression of genes that are specifically related to antibiotic resistance (i.e., that encode proteins that are involved in the molecular-level resistance of the bacteria to the drug). Thus, we took the set of genes that were upregulated in *S. equorum* strain KM031 (not DEGs) in response to any of the antibiotics and subtracted genes detected in the two C^S^E^S^L^S^ strains. This left 65 strain KM1031-specific genes (Table 1). The *msr* (AWC34_RS11115) gene was among them. In a previous study, we suggested that an antibiotic biosynthesis monooxygenase-encoding gene (*abm* AWC34_RS01805) and a lincosamide nucleotydyltransferase-encoding gene (*lnuA,* AWC34_RS13300) might confer resistance to chloramphenicol and lincomycin, respectively^13^. These genes were also among the KM1031-specific genes and *abm* and *lnuA* were slightly upregulated by exposure to chloramphenicol and lincomycin, respectively (Table 1). The lincomycin-resistance phenotype of *lnuA* in strain KM1031 has already been reported^22^. These results imply that *abm* may confer resistance to chloramphenicol, although *abm* was not significantly upregulated by chloramphenicol treatment.

**Table 1:** Expression of *S. equorum* KM1031-specific genes (identified by comparative genomic analysis) following treatment with chloramphenicol, erythromycin or lincomycin.

Gene locus	Product	Log_2_ (fold-change)^a^			COG	Localization
		CHL	ERY	LIN		
AWC34_RS00560	Melibiose:sodium transporter MelB	0.88	− 0.78	− 1.37	G	Chromosome
AWC34_RS00565	Alpha-glucosidase/alpha-galactosidase	1.78	0.13	− 1.28	G	Chromosome
AWC34_RS00570	AraC family transcriptional regulator	− 1.03	− 0.92	− 1.59	K	Chromosome
AWC34_RS01535	UDP-glucose 4-epimerase	− 0.13	− 2.01	0.16	M	Chromosome
AWC34_RS01540	Capsular biosynthesis protein	0.44	− 1.69	− 0.32	M	Chromosome
AWC34_RS01545	UDP-*N*-acetylglucosamine 2-epimerase (non-hydrolyzing)	0.92	− 2.09	0.12	M	Chromosome
AWC34_RS01550	Acetyltransferase	− 0.70	− 1.86	0.46	R	Chromosome
AWC34_RS01555	Capsular biosynthesis protein	− 0.29	− 2.59	0.07	M	Chromosome
AWC34_RS01560	O-antigen ligase family protein	− 0.88	− 1.91	− 1.86	M	Chromosome
AWC34_RS01565	Capsular biosynthesis protein	− 1.68	− 2.31	− 1.33	M	Chromosome
AWC34_RS01570	Nucleotide sugar dehydrogenase	− 0.07	− 2.14	− 0.51	M	Chromosome
AWC34_RS01575	Glycosyltransferase WbuB	− 0.01	− 1.13	− 0.44	M	Chromosome
AWC34_RS01585	Hypothetical protein	− 0.75	− 2.84	− 1.01	M	Chromosome
AWC34_RS01595	LytR family transcriptional regulator	− 0.23	− 0.10	− 0.22	K	Chromosome
AWC34_RS01660	Hypothetical protein	− 3.09	− 0.64	− 1.06	G	Chromosome
AWC34_RS01800	IS6-like element IS257 family transposase	0.28	0.69	− 0.89	L	Chromosome
AWC34_RS01805	Antibiotic biosynthesis monooxygenase	0.62	− 0.06	− 0.39	S	Chromosome
AWC34_RS01870	HTH domain-containing protein	− 1.25	− 0.93	− 0.03	L	Chromosome
AWC34_RS01875	Hypothetical protein	− 1.10	− 0.44	− 0.64	PR	Chromosome
AWC34_RS01880	Hypothetical protein	− 1.63	0.30	0.52	SPO	Chromosome
AWC34_RS01885	Metal-sensitive transcriptional regulator	− 3.18	− 2.78	0.74	S	Chromosome
AWC34_RS01890	Sulfite exporter TauE/SafE family protein	− 3.39	− 0.62	− 0.40	S	Chromosome
AWC34_RS01965	ABC transporter ATP-binding protein	− 0.08	− 0.22	− 1.02	Q	Chromosome
AWC34_RS01970	ABC transporter permease	− 0.85	− 1.83	− 1.45	V	Chromosome
AWC34_RS01975	Formate dehydrogenase	0.17	− 0.75	2.62	C	Chromosome
AWC34_RS02005	LLM class flavin-dependent oxidoreductase	0.78	1.86	− 0.54	C	Chromosome
AWC34_RS02010	Ribosomal-processing cysteine protease Prp	0.22	0.12	− 0.07	J	Chromosome
AWC34_RS03190	CHAP domain-containing protein	− 0.21	1.80	1.34	S	Chromosome
AWC34_RS03985	Arsenate reductase (thioredoxin)	− 1.12	1.83	− 0.16	T	Chromosome
AWC34_RS10815	Transcriptional regulator	− 1.76	− 1.24	0.38	K	Chromosome
AWC34_RS10880	Hypothetical protein	− 0.56	− 1.95	1.02	G	Chromosome
AWC34_RS11115	Msr family ABC-F type ribosomal protection protein	− 0.38	4.88	0.33	R	Chromosome
AWC34_RS11790	Hypothetical protein	0.88	2.20	0.42	S	Chromosome
AWC34_RS12575	Type I restriction endonuclease subunit R	1.01	− 0.38	− 0.76	V	Chromosome
AWC34_RS12585	Restriction endonuclease subunit S	− 0.13	− 1.48	− 0.93	V	Chromosome
AWC34_RS12605	Site-specific DNA-methyltransferase	0.32	− 0.95	− 0.02	SL	Chromosome
AWC34_RS12610	ApaLI family restriction endonuclease	0.28	− 0.90	− 0.30	V	Chromosome
AWC34_RS12660	Hypothetical protein	1.28	− 1.79	− 1.72	V	Chromosome
AWC34_RS12665	DUF2357 domain-containing protein	− 0.93	− 2.09	− 1.03	S	Chromosome
AWC34_RS13330	50S Ribosomal protein L33	− 0.12	0.75	0.09	S	Chromosome
AWC34_RS13395	Hypothetical protein	1.03	− 0.02	0.44	S	Chromosome
AWC34_RS12845	Recombinase family protein	− 2.18	− 0.54	0.17	L	Plasmid1
AWC34_RS12865	Putative sulfate exporter family transporter	− 0.26	1.82	0.37	S	Plasmid1
AWC34_RS12925	Hypothetical protein	0.22	1.06	− 1.01	S	Plasmid1
AWC34_RS12980	Hypothetical protein	− 1.60	− 0.90	− 0.01	L	Plasmid1
AWC34_RS12985	Crp/Fnr family transcriptional regulator	− 0.15	0.12	0.28	T	Plasmid1
AWC34_RS12990	DNA starvation/stationary phase protection protein	0.05	0.06	0.19	P	Plasmid1
AWC34_RS12995	Copper chaperone	− 0.08	− 0.97	− 0.02	P	Plasmid1
AWC34_RS13000	Heavy metal translocating P-type ATPase	0.74	1.50	− 0.60	P	Plasmid1
AWC34_RS13035	Hypothetical protein	1.40	0.37	− 1.55	S	Plasmid1
AWC34_RS13115	XRE family transcriptional regulator	0.06	1.04	1.17	K	Plasmid2
AWC34_RS13160	MurR/RpiR family transcriptional regulator	− 0.13	− 0.83	− 0.33	K	Plasmid2
AWC34_RS13165	Betaine-aldehyde dehydrogenase	− 1.64	− 2.65	− 0.86	KC	Plasmid2
AWC34_RS13170	4-Hydroxy-tetrahydrodipicolinate synthase	− 0.97	− 1.68	− 0.91	E	Plasmid2
AWC34_RS13175	FAD-dependent oxidoreductase	− 0.67	− 0.58	− 0.70	H	Plasmid2
AWC34_RS13180	Hypothetical protein	− 1.29	− 1.48	− 1.08	S	Plasmid2
AWC34_RS13185	SDR family NAD(P)-dependent oxidoreductase	0.31	− 0.27	− 0.42	R	Plasmid2
AWC34_RS13190	LysR family transcriptional regulator	− 0.10	− 1.07	− 0.17	K	Plasmid2
AWC34_RS13195	Acetylornithine deacetylase	0.45	1.79	0.07	E	Plasmid2
AWC34_RS13215	LysE family translocator	− 1.69	− 1.67	− 1.85	E	Plasmid2
AWC34_RS13220	Recombinase family protein	− 0.68	0.61	− 0.23	L	Plasmid2
AWC34_RS13280	Threonine/serine exporter	0.14	1.05	− 0.58	S	Plasmid2
AWC34_RS13285	Threonine/serine exporter	− 0.25	− 0.66	0.03	S	Plasmid2
AWC34_RS13295	Protein rep	− 2.60	− 0.86	− 1.10	L	pSELNU1
AWC34_RS13300	Lincosamide nucleotidyltransferase Lnu(A)'	0.02	− 1.16	0.54	S	pSELNU1

Control indicates strain KM1031 cultured without antibiotics.

*CHL* chloramphenicol, *ERY* erythromycin, *LIN* lincomycin, *COG* clusters of orthologous groups.

^**a**^[Fold-change] was defined as [TMM_antibiotic_/TMM_control_]. Values are means from duplicate experiments.

**Figure 4:**
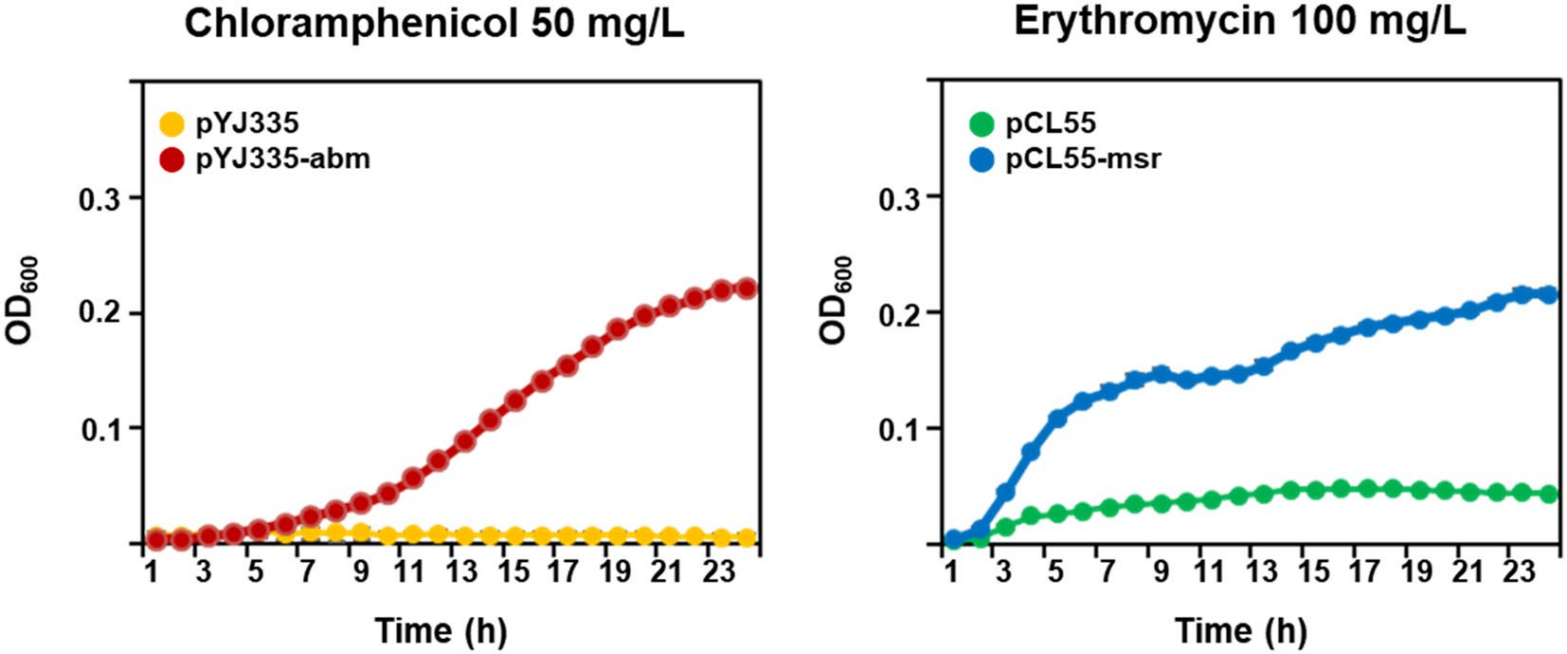
Effect of overexpression of *S. equorum* strain KM1031 genes *abm* and *msr* on resistance of *Escherichia coli* to chloramphenicol and erythromycin, respectively.

To investigate the effect of the *abm* and *msr* genes on chloramphenicol and erythromycin resistance, the genes AWC34_RS01805 and AWC34_RS11115 were PCR amplified and then cloned into the pYJ335 and pCL55 vectors, respectively. The resulting plasmids were designated pYJ335-abm for the gene AWC34_RS01805 and pCL55-msr for the gene AWC34_RS11115. *E. coli* transformants harboring pYJ335-abm and pCL55-msr grew under chloramphenicol and erythromycin pressure, respectively (Fig. 4). Collectively, these results suggested that chloramphenicol and erythromycin treatment modified the expression of the *abm* and *msr* genes in *S. equorum* strain KM031, and that the gene products encoded by these genes contributed to the phenotypic resistance of *E. coli* cells to these antibiotics.

### Effects of antibiotics on two-component systems

Although we identified specific genes that may be responsible for the observed antibiotic resistance of *S. equorum* strain KM031, the transcriptional regulators of specific antibiotic resistance gene expression remained unclear. Two-component systems (TCSs) are the most common systems for bacterial signal transduction in response to environmental signals such as antibiotics and salts. TCS signaling is involved in bacterial resistance to antibiotics. Several TCSs have been detected in *S. equorum* genomes^13^. However, most TCS genes were not markedly up- or down-regulated in our experiments, except the WalKR TCS (Table 2). Although *walKR* genes were not significantly differentially expressed, expression of these genes increased following treatment with chloramphenicol, erythromycin, and lincomycin. The WalKR TCS regulates genes responsible for cell wall metabolism and homeostasis, as well as genes involved in stress responses, virulence, and biofilm formation^23–26^. Although the WalR consensus binding site (5′-TGTWAH N_5_ TGTWAH-3′)^27^ was not identified upstream of *abm*, *msr* or *lnuA*, we hypothesize that the WalKR TCS might be related to expression of these three antibiotic-specific responsive genes.

**Table 2:** Effects of antibiotics on expression of genes related to two-component systems.

Gene locus	Gene	Product	Log_2_ (fold-change)^a^			COG
			CHL	ERY	LIN	
AWC34_RS05855	*arlS*	Sensor histidine kinase	0.66	− 0.35	− 0.24	T
AWC34_RS05860	*arlR*	Response regulator	− 0.58	− 0.66	0.8	T
AWC34_RS07115	*ciaH*	Sensor histidine kinase	0.4	0.49	− 0.17	T
AWC34_RS07120	*phoP*	Response regulator	− 0.13	0.32	− 0.41	T
AWC34_RS07785	*yhcY*	Sensor histidine kinase	− 0.01	− 0.27	0.32	T
AWC34_RS07780	*nreC*	Response regulator	− 0.08	− 0.44	0.89	K
AWC34_RS08090	*vraS*	Sensor histidine kinase	− 0.64	− 0.56	0.07	T
AWC34_RS08085	*vraS*	Response regulator	0.11	0.22	− 0.07	T
AWC34_RS09910	*hssS*	Sensor histidine kinase	0.16	0.64	− 0.42	T
AWC34_RS09905	*afsQ1*	Response regulator	− 0.09	0.6	− 0.23	C
AWC34_RS10085	*nreB*	Sensor histidine kinase	− 2.4	− 1.42	− 0.96	T
AWC34_RS10080	*nreC*	Response regulator	− 2.64	− 1.03	− 1.11	K
AWC34_RS12210	*arlS*	Sensor histidine kinase	− 0.27	− 0.18	0.9	T
AWC34_RS12205	*arlR*	Response regulator	− 1.23	0.16	0.51	T
AWC34_RS12520	*dcuS*	Sensor histidine kinase	0.22	− 0.66	− 0.23	T
AWC34_RS12515	*dcuR*	Response regulator	0.63	− 0.26	− 0.76	T
AWC34_RS12690	*walK*	Sensor histidine kinase	1.13	0.68	0.34	T
AWC34_RS12695	*walR*	Response regulator	1.03	0.25	0.32	T

Control indicates strain KM1031 cultured without antibiotics.

*CHL* chloramphenicol, *ERY* erythromycin, *LIN* lincomycin, *COG* clusters of orthologous groups.

^a^[Fold-change] was defined as [TMM_antibiotic_/TMM_control_]. Values are means from duplicate experiments.

### Validation of RNA-Seq data by qRT-PCR

**Figure 5:**
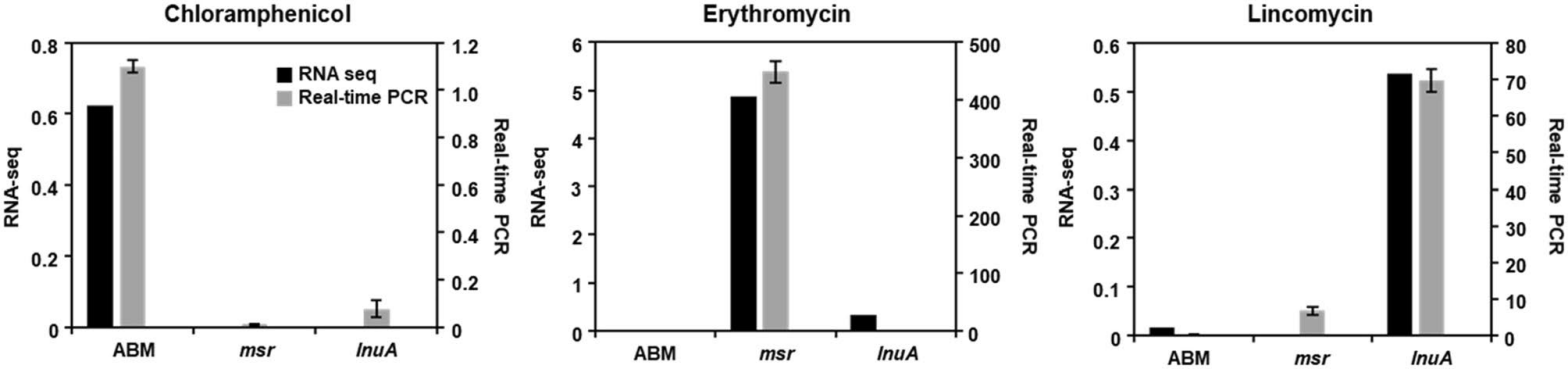
Validation of RNA sequencing data by quantitative real-time PCR (qRT-PCR). Genes related to resistance to chloramphenicol, erythromycin, and lincomycin were selected for validation under different antibiotic pressures. Data are expressed as log_2_ fold-changes in gene expression between control and antibiotic-treated samples. In qRT-PCR, 16S rRNA gene expression was used for normalization of target gene expression.

qRT-PCR was used to validate the *S. equorum* strain KM031 transcriptional profiles obtained by RNA-Seq analysis. As shown in Fig. 5, the expression patterns for each gene (*abm*, *msr*, and *lnuA*) were similar by qRT-PCR and RNA-Seq. Expression of the *abm*, *msr* and *lnuA* genes increased following exposure to chloramphenicol, erythromycin, and lincomycin, respectively. In addition, expression of *walKR* genes was increased following exposure the three antibiotics, although not significantly (Supplementary Fig. S3). Thus, our RNA-Seq data were confirmed by qRT-PCR.

## Discussion

We sought to identify the genes that confer resistance to the antibiotics chloramphenicol, erythromycin and lincomycin in *S. equorum* strain KM031. Using transcriptomic analysis, we confirmed that *abm*, *msr*, and *lnuA* are associated with resistance to chloramphenicol, erythromycin, and lincomycin, respectively.

Chloramphenicol binds to residues A2451 and A2452 of the 23S rRNA and inhibits peptidyl transferase activity by hampering the binding of transfer RNA to the A site of the ribosome^28^. The most common mechanism of resistance to chloramphenicol in bacteria is enzymatic inactivation of chloramphenicol by acetylation, mainly via acetyltransferases, or, in some cases, by chloramphenicol phosphotransferases. Antibiotic biosynthesis monooxygenase oxidizes phenolic compounds and acts as a superoxide scavenger as a defense mechanism in the host^29^. It is also involved in dehalogenation of various aromatic and aliphatic compounds^30^. Chloramphenicol is a halogen and phenyl-containing antibiotic. Therefore, we suggest that antibiotic biosynthesis monooxygenase (*abm*, AWC34_RS01805) might contribute to the loss of chloramphenicol activity in *S. equorum* strain KM031 through phenol-oxidation and/or dehalogenation. This hypothesis requires further study.

Erythromycin inhibits protein synthesis and subsequent structural and functional processes by binding to the 23S rRNA^31^. Erythromycin interferes with aminoacyl translocation, preventing the transfer of the tRNA bound at the A site of the rRNA complex to the P site of the rRNA complex, and consequently prevents movement along mRNA. The most common mechanisms of resistance to erythromycin in bacteria are target modification through methylation of 23S rRNA catalyzed by the product of the *erm* gene and efflux of erythromycin^31,32^. Some reports suggest that *msr* encodes an antibiotic ABC transporter ATP-binding protein and confers resistance to erythromycin through energy-dependent efflux of erythromycin^21,33^. However, Sharkey et al.^34^ suggested that Msr confers erythromycin resistance through ribosomal protection. Strain KM1031 possesses, strain-specifically, an *msr* (AWC34_RS11115) gene. The product of the *msr* gene contains three conserved motifs: Walker A (GXXGXGKST), Walker B (hhhhDEPTNXLD, where h is a hydrophobic residue), and the signature motif (LSGGE)^35,36^. Transmembrane prediction software (TMHMM Serve v.2.20) did not predict a transmembrane domain in the MsrA of *S. equorum* strain KM1031. Therefore, we suggest that *S. equorum* strain KM1031Msr might confer erythromycin resistance through ribosomal protection, rather than by acting as an efflux pump.

Lincomycin belongs to the lincosamides, which interact with the A and P sites of the 50S ribosome^37^. Although the binding sites of the lincosamides differ from those of chloramphenicol, lincosamides also inhibit protein synthesis by inhibition of peptidyl transferases^38^. The most common mechanisms of resistance to lincomycin in bacteria are enzymatic inactivation by acetylation, mainly via acetyltransferases, and efflux^39^. LnuA modifies lincomycin by AMP addition onto the hydroxyl groups of the methylthiolincosamide via nucleotidyltransferase reaction^40^. Strain KM1031 possesses a lincosamide nucleotidyltransferase-encoding gene (*lnuA*; AWC34_RS13300), and thus we assume that LnuA confers resistance to lincomycin via lincomycin modification.

Ahmad et al. suggested that antibiotic resistance genes may be regulated by TCSs^41^. However, we found that TCSs were slightly upregulated, although not significantly, by antibiotic treatment in this study. Thus, if TCSs are involved in the expression of antibiotic resistance genes in strain KM1031, their activity may be regulated by phosphorylation, rather than by changes in their expression levels following antibiotic exposure. The expression of the *walKR* TCS was shown to increase slightly in *S. equorum* strain KM1031 under antibiotic pressure both by RNA-Seq and RT-PCR analyses (Table 2; and Supplementary Fig. S3). Although we did not identify the DNA binding motif of WalR upstream of the three putative antibiotic resistance genes reported in this study, we suggest that the *walKR* TCS may be involved in expression of antibiotic resistance genes.

Apart from TCSs, global regulators may be involved in the expression of antibiotic-specific response genes. Using the keyword “regulator,” 138 regulator genes were detected in the genome of *S. equorum* KM1031 (Supplementary Table S9); 10 and two of these genes were significantly upregulated by erythromycin and lincomycin, respectively. Prior results suggested that such regulators are involved in the expression of antibiotic resistance genes, but the underlying mechanism remains unclear^42,43^. Because regulators bind to their targets as dimers, we checked for direct repeat sequences upstream of *abm*, *msr*, and *lnuA* using Tandem Repeats Finder^44^, but no repeats or palindromes were identified. Additional studies will be required to understand the regulation of antibiotic resistance-related gene expression in *S. equorum* KM1031.

Generally, the molecular mechanisms through which bacteria become drug resistant involve antibiotic efflux, antibiotic inactivation, or alteration of the antibiotic target site in the bacterium. We suggest that alteration of chloramphenicol and lincomycin activity by the products of the *abm* and *lnuA* genes, respectively, contribute to the resistance of *S. equorum* strain KM1031 to these antibiotics, and that *msr* confers the erythromycin resistance of strain KM1031 by ribosomal protection. Changes in expression of genes related to efflux, transport, and salt tolerance may nonspecifically contribute to resistance to all three antibiotics. However, further studies are required to define the specific mechanisms of resistance, including gene regulation and the mechanism of acquisition of relevant genes. This information will help reduce the antibiotic resistance of food bacteria such as starters involved in food fermentation.

## Supplementary Information

Supplementary Information 1.

Supplementary Information 2.

## Data Availability

RNA-Seq data analyzed in this study were deposited in the Sequence Read Archive (SRR10807062–SRR10807065). The data presented in this study are available in the article/Supplementary Material; further inquiries can be directed to the corresponding author.
