# Supplementary material for: Transcriptomic analysis of Staphylococcus equorum KM1031 from the high-salt fermented seafood jeotgal under chloramphenicol, erythromycin and lincomycin stresses
Source: Sci Rep. 2022 Sep 15;12:15541. doi: 10.1038/s41598-022-19897-9 (PMC9477809; doi:10.1038/s41598-022-19897-9)
Supplement: Supplementary file 1 — Supplementary Information 1. [file 41598_2022_19897_MOESM1_ESM.docx]

**Supplementary Table 1.** Number of sequence reads in RNA-Seq.

|  | **Control** | **15 mg/l Chloramphenicol** | **5 ml/l Erythromycin** | **30 mg/l Lincomycin** |
| --- | --- | --- | --- | --- |
| **rRNA reads** | 199,382 | 408,586 | 713,181 | 150,726 |
| **(%)** | 0.67 | 1.17 | 1.56 | 0.38 |
| **mRNA reads** | 24,701,434 | 28,965,916 | 37,661,928 | 32,409,376 |
| **(%)** | 83.01 | 82.89 | 82.63 | 82.40 |
| **Intergenic reads** | 3,854,652 | 4,542,236 | 5,735,230 | 4,956,787 |
| **(%)** | 12.95 | 13.00 | 12.58 | 12.60 |
| **Unmapped reads** | 838,262 | 913,698 | 1,345,032 | 1,287,556 |
| **(%)** | 2.82 | 2.61 | 2.95 | 3.27 |
| **QC dropped reads** | 164,029 | 115,989 | 125,818 | 528,920 |
| **(%)** | 0.55 | 0.33 | 0.28 | 1.34 |
| **Total reads** | 29,757,759 | 34,946,425 | 45,581,189 | 39,333,365 |
| **(%)** | 96.6 | 97.1 | 96.8 | 95.4 |

**Supplementary Table 3**. Comparison of normalization methods for RNA-Seq.

| **Normalization method** | | | |
| --- | --- | --- | --- |
| **Coefficients of variation (CV)** | **RPKM** | **RLE** | **TMM** |
|  | 0.6576 | 0.4976 | 0.4982 |

**Supplementary Table 4.** Primers for quantitative real-time PCR.

| **Gene** | **Locus** | **Oligonucleotide** |  | **Size** | **Reference** |
| --- | --- | --- | --- | --- | --- |
|  |  | **Forward (5´→3´)** | **Reverse (5´→3´)** | **(bp)** |  |
| 16S rRNA | AWC34_RS01040 | ACT CCT ACG GGA GGC AGC AGT | TAT TAC CGC GGC TGC TGG C | 198 | Clifford et al. 2012 |
| *sodA* | AWC34_RS06475 | GTG GAG GAC ACT TAA ACC ATT C | CAA TTT ACC ATC GTT TAC AAC TAG | 193 | Blaiotta et al. 2004 |
| *abm* | AWC34_RS01805 | CCG TCA ATG CCG CAG CCA C | CCT ACG TGG GGC CCT ACC | 158 | This study |
| *msr* | AWC34_RS11115 | GGA GCG GGA TAC CTT CGC G | GCA GCA CAG AAA CAG GCG | 175 | This study |
| *lnuA* | AWC34_RS13300 | CCA TAC GTG AAG GCA TCC | GGA GGC TGG GGC CTA GAT C | 154 | This study |

Clifford, R.J., Milillo, M., Prestwood, J., Quintero, R., Zurawski, D.V., Kwak, Y.I., et al. (2012). Detection of bacterial 16S rRNA and identification of four clinically important bacteria by real-time PCR. *PLoS ONE* 7:e48558. doi: 10.1371/journal.pone.0048558

Blaiotta, G., Ercolini, D., Mauriello, G., Salzano, G., Villani, F. (2004). Rapid and reliable identification of *Staphylococcus equorum* by a species-specific PCR assay targeting the *sod*A gene. Systematic and Applied Microbiology, 27: 696-702

**Supplementary Table 5**. Primers for cloning of abm and msr genes into plasmid.

| **Gene** | **Locus** | **Oligonucleotide** |  | **Size** | **Reference** |
| --- | --- | --- | --- | --- | --- |
|  |  | **Forward (5´→3´)** | **Reverse (5´→3´)** | **(bp)** |  |
| *abm* | AWC34_RS01805 | CCG CTC GAG CGG CTA CAG TGA TAA CAT CC | CCG CTC GAG CGG ATT TTC GCC ATG CCA CG | 756 | This study |
| *msr* | AWC34_RS11115 | GGA ATT CAG TGA GTG AGA TAG GAG AA A CC | GGA ATT CAC CCC CAA TAC TTC TCT ACC ATC | 1,591 | This study |

Underlined sequences is the restriction enzyme sites.

**Supplementary Table 6.** Summary of genes up-regulated by chloramphenicol, erythromycin and lincomycin.

| **Gene locus** | **Product** | **COG** | **Log2 (Fold change)** | | | |  | **Comparative** | | |
| --- | --- | --- | --- | --- | --- | --- | --- | --- | --- | --- |
|  |  |  | **CHL** | **ERY** | | **LIN** |  | **KM1031** | **C2014** | **KS1039** |
| **Translation, ribosomal structure and biogenesis** | | | | | | |  |  |  |  |
| AWC34_RS00005 | 50S ribosomal protein L34 | J | -0.41 | 2.52 | | -0.16 |  | ● | ● | ● |
| AWC34_RS00610 | 30S ribosomal protein S6 | J | 0.93 | 3.93 | | 2.09 |  | ● | ● | ● |
| AWC34_RS00620 | 30S ribosomal protein S18 | J | 0.42 | 2.28 | | 1.69 |  | ● | ● | ● |
| AWC34_RS01170 | 50S ribosomal protein L25 | J | 1.29 | 3.43 | | 0.4 |  | ● | ● | ● |
| AWC34_RS01250 | Lysine--tRNA ligase | J | 0.68 | 2.14 | | -0.42 |  | ● | ● | ● |
| AWC34_RS01430 | 50S ribosomal protein L11 | J | 0.91 | 2.97 | | -0.45 |  | ● | ● | ● |
| AWC34_RS01435 | 50S ribosomal protein L1 | J | 1.37 | 2.84 | | -0.95 |  | ● | ● | ● |
| AWC34_RS01440 | 50S ribosomal protein L10 | J | 0.82 | 3.82 | | 0.41 |  | ● | ● | ● |
| AWC34_RS01445 | 50S ribosomal protein L7/L12 | J | 0.82 | 3.42 | | -0.98 |  | ● | ● | ● |
| AWC34_RS01450 | Class I SAM-dependent methyltransferase | J | 0.5 | 4.23 | | 0.25 |  | ● | ● | ● |
| AWC34_RS01470 | 30S ribosomal protein S12 | J | 0.17 | 2.23 | | 0.58 |  | ● | ● | ● |
| AWC34_RS01475 | 30S ribosomal protein S7 | J | 0.37 | 2.27 | | -0.04 |  | ● | ● | ● |
| AWC34_RS01480 | Elongation factor G | J | 0.73 | 2.44 | | -0.34 |  | ● | ● | ● |
| AWC34_RS01485 | Elongation factor Tu | J | 0.9 | 2.3 | | -0.6 |  | ● | ● | ● |
| AWC34_RS01505 | GNAT family N-acetyltransferase | J | -0.77 | 2.39 | | -0.34 |  | ● | ● | ● |
| AWC34_RS03890 | Hypothetical protein | J | 0.72 | -0.1 | | 2.26 |  | ● | ● | ● |
| AWC34_RS04420 | 50S ribosomal protein L32 | J | -0.69 | 2.51 | | -0.65 |  | ● | ● | ● |
| AWC34_RS04805 | 50S ribosomal protein L28 | J | 0.11 | 2.24 | | -0.84 |  | ● | ● | ● |
| AWC34_RS04915 | 50S ribosomal protein L19 | J | 1.4 | 4.58 | | -0.02 |  | ● | ● | ● |
| AWC34_RS04985 | 30S ribosomal protein S2 | J | 0.92 | 4.95 | | 0.25 |  | ● | ● | ● |
| AWC34_RS04990 | Elongation factor Ts | J | 0.64 | 2.83 | | -0.36 |  | ● | ● | ● |
| AWC34_RS05080 | 30S ribosomal protein S15 | J | -0.12 | 3.16 | | 0.98 |  | ● | ● | ● |
| AWC34_RS06585 | 30S ribosomal protein S21 | J | 2.54 | 2.98 | | 2.91 |  | ● | ● | ● |
| AWC34_RS06785 | tRNA 2-thiouridine(34) synthase MnmA | J | 0.21 | 1.89 | | 3.25 |  | ● | ● | ● |
| AWC34_RS06910 | 50S ribosomal protein L27 | J | 0.26 | 3.1 | | 0.53 |  | ● | ● | ● |
| AWC34_RS06915 | Ribosomal-processing cysteine protease Prp | J | 0.89 | 3.85 | | 2.21 |  | ● | ● | ● |
| AWC34_RS06920 | 50S ribosomal protein L21 | J | 1.15 | 4.08 | | 0.79 |  | ● | ● | ● |
| AWC34_RS07055 | 50S ribosomal protein L20 | J | 1.25 | 3.22 | | 0.22 |  | ● | ● | ● |
| AWC34_RS07060 | 50S ribosomal protein L35 | J | 1.13 | 3.67 | | -1.05 |  | ● | ● | ● |
| AWC34_RS07065 | Translation initiation factor IF-3 | J | 0.57 | 3.23 | | 0.18 |  | ● | ● | ● |
| AWC34_RS07265 | 30S ribosomal protein S4 | J | -0.18 | 2.83 | | -0.69 |  | ● | ● | ● |
| AWC34_RS07450 | Leucine--tRNA ligase | J | 2 | 0.16 | | -0.5 |  | ● | ● | ● |
| AWC34_RS08105 | Type I methionyl aminopeptidase | J | 0.67 | -0.04 | | 2.1 |  | ● | ● | ● |
| AWC34_RS08770 | Type B 50S ribosomal protein L31 | J | -0.01 | 2.26 | | 1.4 |  | ● | ● | ● |
| AWC34_RS09160 | 30S ribosomal protein S9 | J | 0.27 | 3.04 | | -0.38 |  | ● | ● | ● |
| AWC34_RS09165 | 50S ribosomal protein L13 | J | 0.34 | 3.41 | | 0.23 |  | ● | ● | ● |
| AWC34_RS09170 | tRNA pseudouridine(38-40) synthase TruA | J | -0.36 | 3.22 | | 0.38 |  | ● | ● | ● |
| AWC34_RS09190 | 50S ribosomal protein L17 | J | 1.48 | 4.08 | | -0.2 |  | ● | ● | ● |
| AWC34_RS09200 | 30S ribosomal protein S11 | J | 1.86 | 4.04 | | 0.71 |  | ● | ● | ● |
| AWC34_RS09205 | 30S ribosomal protein S13 | J | 2.11 | 3.79 | | 0.98 |  | ● | ● | ● |
| AWC34_RS09210 | 50S ribosomal protein L36 | J | 1.71 | 2.23 | | 1.71 |  | ● | ● | ● |
| AWC34_RS09215 | Translation initiation factor IF-1 | J | 2.24 | 2.6 | | -0.29 |  | ● | ● | ● |
| AWC34_RS09230 | 50S ribosomal protein L15 | J | 2.49 | 4.1 | | 1.14 |  | ● | ● | ● |
| AWC34_RS09235 | 50S ribosomal protein L30 | J | 2.97 | 4.83 | | 0.29 |  | ● | ● | ● |
| AWC34_RS09240 | 30S ribosomal protein S5 | J | 2.42 | 4.15 | | 0.33 |  | ● | ● | ● |
| AWC34_RS09245 | 50S ribosomal protein L18 | J | 2.4 | 3.88 | | 0.99 |  | ● | ● | ● |
| AWC34_RS09250 | 50S ribosomal protein L6 | J | 2.37 | 3.7 | | 0.87 |  | ● | ● | ● |
| AWC34_RS09255 | 30S ribosomal protein S8 | J | 2.5 | 3.83 | | 0.62 |  | ● | ● | ● |
| AWC34_RS09260 | 30S ribosomal protein S14 type Z | J | 2.52 | 4.03 | | 0.81 |  | ● | ● | ● |
| AWC34_RS09265 | 50S ribosomal protein L5 | J | 2.3 | 3.98 | | 0.74 |  | ● | ● | ● |
| AWC34_RS09270 | 50S ribosomal protein L24 | J | 2.31 | 4.39 | | 1.32 |  | ● | ● | ● |
| AWC34_RS09275 | 50S ribosomal protein L14 | J | 2.18 | 4.28 | | 1.3 |  | ● | ● | ● |
| AWC34_RS09280 | 30S ribosomal protein S17 | J | 2 | 2.9 | | 0.72 |  | ● | ● | ● |
| AWC34_RS09285 | 50S ribosomal protein L29 | J | 2.13 | 4.02 | | 0.5 |  | ● | ● | ● |
| AWC34_RS09290 | 50S ribosomal protein L16 | J | 2.06 | 4.28 | | 1.2 |  | ● | ● | ● |
| AWC34_RS09295 | 30S ribosomal protein S3 | J | 1.9 | 3.97 | | 0.77 |  | ● | ● | ● |
| AWC34_RS09300 | 50S ribosomal protein L22 | J | 1.66 | 3.47 | | 1.97 |  | ● | ● | ● |
| AWC34_RS09305 | 30S ribosomal protein S19 | J | 1.99 | 4.15 | | 1.18 |  | ● | ● | ● |
| AWC34_RS09310 | 50S ribosomal protein L2 | J | 1.33 | 3.47 | | 0.99 |  | ● | ● | ● |
| AWC34_RS09315 | 50S ribosomal protein L23 | J | 1.68 | 3.84 | | 1.26 |  | ● | ● | ● |
| AWC34_RS09320 | 50S ribosomal protein L4 | J | 1.68 | 3.48 | | 1.14 |  | ● | ● | ● |
| AWC34_RS09325 | 50S ribosomal protein L3 | J | 1.82 | 3.99 | | 0.93 |  | ● | ● | ● |
| AWC34_RS09330 | 30S ribosomal protein S10 | J | 1.81 | 4.11 | | 0.91 |  | ● | ● | ● |
| AWC34_RS11760 | tRNA-dihydrouridine synthase | J | -0.02 | 2.29 | | -0.38 |  | ● | ● | ● |
| AWC34_RS12075 | RluA family pseudouridine synthase | J | 0.3 | 4.25 | | -0.65 |  | ● | ● | ● |
| **Transcription, regulators** | | |  |  | |  |  |  |  |  |
| AWC34_RS00030 | Chromosome partitioning protein ParB | K | 0.55 | 3.92 | | -0.55 |  | ● | ● | ● |
| AWC34_RS00485 | Transcriptional regulator | K | 1 | 2.61 | | -0.2 |  | ● | ● | ● |
| AWC34_RS00675 | Transcriptional regulator | K | -0.72 | -1.3 | | 2.06 |  | ● | ● | ● |
| AWC34_RS01010 | Trehalose operon repressor | K | 0.65 | 2.18 | | 0.12 |  | ● | ● | ● |
| AWC34_RS01455 | DNA-directed RNA polymerase subunit beta | K | 1.31 | 3.22 | | -0.77 |  | ● | ● | ● |
| AWC34_RS01460 | DNA-directed RNA polymerase subunit beta' | K | 1.32 | 2.94 | | -1.59 |  | ● | ● | ● |
| AWC34_RS02650 | MarR family transcriptional regulator | K | 0.82 | 3.22 | | -1.82 |  | ● | ● | ● |
| AWC34_RS03145 | Ribonuclease R | K | 0.63 | 4.26 | | 1.02 |  | ● | ● | ● |
| AWC34_RS03230 | Cold-shock protein | K | -2.2 | 2.91 | | 0.93 |  | ● | ● | ● |
| AWC34_RS04065 | LytR family transcriptional regulator | K | 0.2 | 2.71 | | 0.16 |  | ● | ● | ● |
| AWC34_RS05745 | Cold-shock protein | K | 0.35 | 0.5 | | 2.01 |  | ● | ● | ● |
| AWC34_RS06205 | Transcriptional repressor | K | -0.18 | 2.37 | | 0.08 |  | ● | ● | ● |
| AWC34_RS06515 | RNA polymerase sigma factor RpoD | K | 0.23 | 3.37 | | 0.44 |  | ● | ● | ● |
| AWC34_RS06730 | Transcription elongation factor GreA | K | 0.39 | 2.07 | | 0.07 |  | ● | ● | ● |
| AWC34_RS07090 | Transcriptional repressor NrdR | K | -0.17 | 0.11 | | 2.15 |  | ● | ● | ● |
| AWC34_RS07755 | Transcriptional regulator | K | -0.4 | 3.78 | | 0.2 |  | ● | ● | ● |
| AWC34_RS08775 | Transcription termination factor Rho | K | 0.69 | 4.27 | | 0.99 |  | ● | ● | ● |
| AWC34_RS09195 | DNA-directed RNA polymerase subunit alpha | K | 1.95 | 4.03 | | 0.54 |  | ● | ● | ● |
| AWC34_RS10350 | TetR/AcrR family transcriptional regulator | K | 0.5 | 2.38 | | 0.63 |  | ● | ● | ● |
| AWC34_RS11110 | MarR family transcriptional regulator | K | -0.5 | 4.19 | | 0.2 |  | ● | ● | ● |
| AWC34_RS11525 | GbsR/MarR family transcriptional regulator | K | -0.03 | 2.09 | | 1.39 |  | ● | ● | ● |
| AWC34_RS11590 | XRE family transcriptional regulator | K | -0.61 | 2.63 | | -0.5 |  | ● | ● | ● |
| AWC34_RS12185 | PadR family transcriptional regulator | K | -1.2 | -0.27 | | 2.27 |  | ● | ● | ● |
| AWC34_RS12270 | Siderophore biosynthesis protein SbnI | K | 2.71 | 3.36 | | 1.2 |  | ● | ● | ● |
| AWC34_RS12705 | MarR family transcriptional regulator | K | -0.79 | 2.97 | | -1.05 |  | ● | ● | ● |
| **Replication, recombination and repair** | | | |  | |  |  |  |  |  |
| AWC34_RS00615 | Single-stranded DNA-binding protein | L | 1.24 | 3.95 | | 2.11 |  | ● | ● | ● |
| AWC34_RS03375 | Topiosmerase | L | -0.17 | -0.47 | | 2.28 |  | ● | ● | ● |
| AWC34_RS04955 | Type I DNA topoisomerase | L | 0.98 | 3.12 | | 0.1 |  | ● | ● | ● |
| AWC34_RS05330 | Thermonuclease | L | -0.35 | 2.43 | | 1.24 |  | ● | ● | ● |
| AWC34_RS05975 | Class I SAM-dependent RNA methyltransferase | L | 0.32 | 2.26 | | 0.6 |  | ● | ● | ● |
| AWC34_RS08015 | A/G-specific adenine glycosylase | L | -0.23 | 2.67 | | 0.36 |  | ● | ● | ● |
| AWC34_RS08585 | ATP-dependent helicase | L | 0.99 | 4.36 | | 0.85 |  | ● | ● | ● |
| AWC34_RS12805 | Chromosomal replication initiator protein DnaA | L | -0.29 | 2.5 | | -1.22 |  | ● | ● | ● |
| **Cell cycle control, cell division, chromosome partitioning** | | | | |  | | | | | |
| AWC34_RS00020 | TRNA uridine-5-carboxymethylaminomethyl(34) synthesis enzyme MnmG | D | 1.21 | 3.84 | | -0.41 |  | ● | ● | ● |
| **Cell wall/membrane/envelope biogenesis** | | | | | |  |  |  |  |  |
| AWC34_RS00025 | 16S rRNA (guanine(527)-N(7))-methyltransferase RsmG | M | 0.86 | 4.07 | | -0.78 |  | ● | ● | ● |
| AWC34_RS00605 | Lysozyme | M | 1.28 | 4.26 | | -0.79 |  | ● | ● | ● |
| AWC34_RS02405 | Glycosyltransferase | M | -0.02 | 2.56 | | -0.34 |  | ● | ● | ● |
| AWC34_RS02435 | DUF1958 domain-containing protein | M | 0.24 | 3.42 | | -0.37 |  | ● | ● | ● |
| AWC34_RS04530 | Glycosyltransferase family 2 protein | M | 2.07 | 1.58 | | -0.19 |  | ● | ● | ● |
| AWC34_RS12435 | UTP--glucose-1-phosphate uridylyltransferase | M | 1.36 | 2.19 | | -0.33 |  | ● |  |  |
| AWC34_RS13385 | CDP-glycerol:glycerophosphateglycerophosphotransferase | M | 1.88 | 2.67 | | -0.62 |  | ● | ● | ● |
| **Post-translational modification, protein turnover, chaperones** | | | | |  | | | | | |
| AWC34_RS01220 | ATP-dependent metallopeptidase FtsH/Yme1/Tma family protein | O | 0.71 | 2.01 | | -0.59 |  | ● | ● | ● |
| AWC34_RS02760 | 7-carboxy-7-deazaguanine synthase QueE | O | 0.49 | 3.57 | | 0.09 |  | ● | ● | ● |
| AWC34_RS03150 | SsrA-binding protein SmpB | O | 0.37 | 4.45 | | 0.99 |  | ● | ● | ● |
| AWC34_RS03615 | Peptidyl-prolyl cis-trans isomerase | O | 0.15 | -0.13 | | 2.07 |  | ● | ● | ● |
| AWC34_RS03805 | Adaptor protein MecA | O | -0.78 | 0.7 | | 2.28 |  | ● | ● | ● |
| AWC34_RS05285 | Glutathione peroxidase | O | 0.54 | 1.19 | | 2.05 |  | ● | ● | ● |
| AWC34_RS08405 | Oxidoreductase | O | 0.15 | 2.15 | | 0.51 |  | ● | ● | ● |
| AWC34_RS11070 | Serine protease | O | 2.12 | 0.69 | | 1.63 |  | ● | ● | ● |
| **Signal transduction mechanisms** | | | |  | |  |  |  |  |  |
| AWC34_RS02955 | GGDEF domain-containing protein | T | -0.35 | 2.14 | | -1.07 |  | ● | ● | ● |
| AWC34_RS04330 | Translational GTPase TypA | T | 1.53 | 2.98 | | -0.24 |  | ● | ● | ● |
| AWC34_RS08540 | STAS domain-containing protein | T | 0.97 | 0.03 | | 2.65 |  | ● | ● | ● |
| AWC34_RS12425 | ATP-binding protein | T | 0.02 | 2.26 | | -0.98 |  | ● |  |  |
| AWC34_RS12970 | Universal stress protein | T | -0.43 | 2.04 | | -1.38 |  | ● | ● | ● |
| **Intracellular trafficking, secretion, and vesicular transport** | | | | |  | | | | | |
| AWC34_RS03135 | Preprotein translocase subunit SecG | U | -0.44 | 2.67 | | 0.95 |  | ● | ● | ● |
| AWC34_RS09225 | Preprotein translocase subunit SecY | U | 2.37 | 3.6 | | 0.35 |  | ● | ● | ● |
| **Defense mechanisms** | |  |  |  | |  |  |  |  |  |
| AWC34_RS11450 | ABC transporter ATP-binding protein | V | 0.56 | 2.01 | | 1.06 |  | ● | ● | ● |
| **Energy production and conversion** | | | |  | |  |  |  |  |  |
| AWC34_RS01975 | Formate dehydrogenase | C | 0.17 | -0.75 | | 2.62 |  | ● |  |  |
| AWC34_RS05470 | Aconitate hydratase AcnA | C | 2.03 | 2.09 | | -0.8 |  | ● | ● | ● |
| AWC34_RS09555 | LLM class flavin-dependent oxidoreductase | C | -0.09 | 2.07 | | 0.2 |  | ● |  | ● |
| AWC34_RS09730 | Sodium:proton antiporter | C | 0 | 3 | | -0.45 |  | ● | ● | ● |
| AWC34_RS11520 | Betaine-aldehyde dehydrogenase | C | 0.42 | 4.32 | | 1.13 |  | ● | ● | ● |
| **Amino acid transport and metabolism** | | | |  | |  |  |  |  |  |
| AWC34_RS00080 | Amino acid permease | E | 0.18 | 3.1 | | -1.05 |  | ● | ● | ● |
| AWC34_RS00495 | Amidohydrolase | E | 2.14 | 1.38 | | -0.57 |  | ● | ● | ● |
| AWC34_RS00940 | Dihydroxy-acid dehydratase | E | 1.29 | 2.06 | | -0.52 |  | ● | ● | ● |
| AWC34_RS00985 | Glutamate synthase large subunit | E | 3.96 | 0.62 | | 0.3 |  | ● | ● | ● |
| AWC34_RS00990 | Glutamate synthase subunit beta | E | 4.67 | 1.68 | | 0 |  | ● | ● |  |
| AWC34_RS01740 | Amino acid permease | E | -0.19 | 2.13 | | -0.75 |  | ● | ● | ● |
| AWC34_RS06790 | Cysteine desulfurase | E | -0.45 | 1.99 | | 4.87 |  | ● | ● | ● |
| AWC34_RS07125 | NADP-dependent isocitrate dehydrogenase | E | 2.36 | 0.96 | | -1.2 |  | ● | ● | ● |
| AWC34_RS07515 | Osmoprotectant ABC transporter substrate-binding protein | E | 1.84 | 3.75 | | 0.99 |  | ● | ● | ● |
| AWC34_RS09760 | Urocanate hydratase | E | 2.23 | 2.25 | | 0.9 |  | ● | ● | ● |
| AWC34_RS10315 | ABC transporter permease | E | 0.41 | 2.07 | | -1.72 |  | ● | ● | ● |
| AWC34_RS10320 | Osmoprotectant ABC transporter substrate-binding protein | E | 0.76 | 2.65 | | -0.53 |  | ● | ● | ● |
| AWC34_RS10325 | ABC transporter permease | E | 0.94 | 2.51 | | -1.3 |  | ● | ● | ● |
| AWC34_RS10385 | FMN-binding glutamate synthase family protein | E | 0.67 | 2.24 | | 0.14 |  | ● | ● | ● |
| AWC34_RS10580 | Sodium/proline symporter PutP | E | 3.12 | 2.47 | | 0.06 |  | ● | ● | ● |
| AWC34_RS11125 | Peptide ABC transporter ATP-binding protein | E | -0.11 | 2.01 | | -0.32 |  | ● | ● | ● |
| AWC34_RS11210 | Cysteine synthase family protein | E | -0.5 | 2.2 | | -0.34 |  | ● | ● | ● |
| AWC34_RS11425 | Acetylglutamate kinase | E | 6.15 | 4.71 | | -0.25 |  | ● | ● | ● |
| AWC34_RS11430 | Bifunctional glutamate N-acetyltransferase/amino-acid acetyltransferase ArgJ | E | 5.78 | 4.61 | | -0.27 |  | ● | ● | ● |
| AWC34_RS11435 | N-acetyl-gamma-glutamyl-phosphate reductase | E | 5.51 | 3.95 | | -0.08 |  | ● | ● | ● |
| AWC34_RS11440 | Ornithine--oxo-acid transaminase | E | 4.49 | 3.98 | | 0.37 |  | ● | ● | ● |
| AWC34_RS11515 | Choline dehydrogenase | E | -0.37 | 4.22 | | 0.42 |  | ● | ● | ● |
| AWC34_RS12275 | Type III PLP-dependent enzyme | E | 2.95 | 3.6 | | 1.56 |  | ● | ● | ● |
| AWC34_RS12305 | 2,3-diaminopropionate biosynthesis protein SbnB | E | 4.04 | 4.08 | | 0.95 |  | ● | ● | ● |
| AWC34_RS12310 | 2,3-diaminopropionate biosynthesis protein SbnA | E | 3.51 | 3.37 | | 0.76 |  | ● | ● | ● |
| AWC34_RS12770 | Histidine ammonia-lyase | E | 2.85 | 1.67 | | 0.48 |  | ● | ● | ● |
| AWC34_RS03780 | ABC transporter ATP-binding protein | EP | 2.61 | 0.68 | | -0.22 |  | ● | ● | ● |
| AWC34_RS11130 | ABC transporter ATP-binding protein | EP | 0.19 | 2.32 | | -0.49 |  | ● | ● | ● |
| **Nucleotide transport and metabolism** | | | |  | |  |  |  |  |  |
| AWC34_RS00365 | Adenosine deaminase | F | 0.26 | 2.24 | | -0.45 |  | ● | ● | ● |
| AWC34_RS00800 | Xanthine phosphoribosyltransferase | F | 1.42 | 3.41 | | 0.58 |  | ● | ● | ● |
| AWC34_RS00805 | Purine permease | F | 2.5 | 3.22 | | 0.11 |  | ● | ● | ● |
| AWC34_RS00815 | Glutamine-hydrolyzing GMP synthase | F | 1.9 | 2.27 | | -0.79 |  | ● | ● | ● |
| AWC34_RS02030 | Deoxynucleoside kinase | F | 0.25 | 3.17 | | -0.39 |  | ● | ● | ● |
| AWC34_RS02035 | Deoxyguanosine kinase | F | -0.09 | 2.94 | | 0.65 |  | ● | ● | ● |
| AWC34_RS02870 | Class Ib ribonucleoside-diphosphate reductase assembly flavoprotein NrdI | F | 0.03 | 1.03 | | 2.17 |  | ● | ● | ● |
| AWC34_RS04130 | Phosphoribosylaminoimidazolesuccinocarboxamidesynthase | F | 2.25 | 0.31 | | 0.49 |  | ● | ● | ● |
| AWC34_RS04135 | Phosphoribosylformylglycinamidine synthase subunit PurS | F | 2.34 | 0.12 | | 0.91 |  | ● | ● | ● |
| AWC34_RS04140 | Phosphoribosylformylglycinamidine synthase I | F | 2.45 | 0.44 | | 0.46 |  | ● | ● | ● |
| AWC34_RS04145 | Phosphoribosylformylglycinamidine synthase subunit PurL | F | 2.86 | 0.95 | | -0.55 |  | ● | ● | ● |
| AWC34_RS04150 | Amidophosphoribosyltransferase | F | 2.61 | 0.98 | | -0.66 |  | ● | ● | ● |
| AWC34_RS04155 | Phosphoribosylformylglycinamidine cyclo-ligase | F | 2.49 | 0.95 | | -0.96 |  | ● | ● | ● |
| AWC34_RS04160 | Phosphoribosylglycinamide formyltransferase | F | 2.45 | 0.89 | | -0.49 |  | ● | ● | ● |
| AWC34_RS04165 | Bifunctionalphosphoribosylaminoimidazolecarboxamideformyltransferase/IMP cyclohydrolase PurH | F | 2.15 | 0.49 | | -0.68 |  | ● | ● | ● |
| AWC34_RS07760 | Formyltetrahydrofolate deformylase | F | 0.72 | 0.7 | | 2.2 |  | ● | ● | ● |
| AWC34_RS09220 | Adenylate kinase | F | 2.54 | 4.25 | | -0.04 |  | ● | ● | ● |
| **Carbohydrate transport and metabolism** | | | | | |  |  |  |  |  |
| AWC34_RS02580 | MFS transporter | G | -0.65 | 3.66 | | 0.52 |  | ● | ● | ● |
| AWC34_RS10200 | MFS transporter | G | 0.13 | 2.71 | | 0.28 |  | ● | ● | ● |
| AWC34_RS10925 | Intercellular adhesion protein C | G | 1.45 | 4.23 | | -0.59 |  | ● | ● | ● |
| AWC34_RS10945 | Transcriptional regulator | G | 0.86 | 2.4 | | -0.35 |  | ● | ● | ● |
| AWC34_RS11470 | MFS transporter | G | 2.83 | 2.07 | | 1.69 |  | ● | ● | ● |
| AWC34_RS11585 | MFS transporter | G | -0.56 | 2.06 | | -0.02 |  | ● | ● | ● |
| AWC34_RS12280 | Siderophore biosynthesis protein SbnG | G | 3.16 | 3.98 | | 1.85 |  | ● | ● | ● |
| AWC34_RS12295 | MFS transporter | G | 3 | 3.59 | | 0.71 |  | ● | ● | ● |
| AWC34_RS12700 | MFS transporter | G | 0.32 | 2.86 | | -0.9 |  | ● | ● | ● |
| AWC34_RS04050 | Autolysin | GM | 2.41 | 1.15 | | -0.27 |  | ● | ● | ● |
| **Coenzyme transport and metabolism** | | | |  | |  |  |  |  |  |
| AWC34_RS01245 | 2-amino-4-hydroxy-6-hydroxymethyldihydropteridine diphosphokinase | H | 0.16 | 2.35 | | 0.08 |  | ● | ● | ● |
| AWC34_RS02765 | 6-carboxytetrahydropterin synthase QueD | H | 0.44 | 3.28 | | 0.28 |  | ● | ● |  |
| AWC34_RS07490 | 6,7-dimethyl-8-ribityllumazine synthase | H | 2.67 | 0.44 | | 0.07 |  | ● | ● | ● |
| AWC34_RS07495 | Bifunctional3,4-dihydroxy-2-butanone-4-phosphate synthase/GTP cyclohydrolase II | H | 3.17 | 0.49 | | -0.03 |  | ● | ● | ● |
| AWC34_RS07500 | Riboflavin synthase | H | 3.22 | 0.49 | | -0.51 |  | ● | ● | ● |
| AWC34_RS07505 | Bifunctionaldiaminohydroxyphosphoribosylaminopyrimidinedeaminase/5-amino-6-(5-phosphoribosylamino)uracilreductase RibD | H | 2.93 | 0.66 | | -0.41 |  | ● | ● | ● |
| AWC34_RS09420 | Molybdopterin converting factor subunit 1 | H | -0.38 | -0.15 | | 2.08 |  | ● | ● | ● |
| AWC34_RS12415 | Biotin synthase BioB | H | 2.09 | -0.54 | | -0.44 |  | ● | ● | ● |
| **Lipid transport and metabolism** | | |  |  | |  |  |  |  |  |
| AWC34_RS00240 | Long-chain fatty acid--CoA ligase | I | 2.58 | 1.04 | | 0.26 |  | ● | ● | ● |
| AWC34_RS00250 | 3-hydroxyacyl-CoA dehydrogenase | I | 2.16 | 0.89 | | 0.48 |  | ● | ● | ● |
| AWC34_RS03260 | Alpha/beta hydrolase | I | 0.66 | -1.63 | | 2.38 |  | ● | ● | ● |
| AWC34_RS10340 | 3-hydroxybutyryl-CoA dehydrogenase | I | -0.19 | 4.2 | | 0.08 |  | ● | ● | ● |
| AWC34_RS00135 | 3-oxoacyl-ACP reductase | IQ | 2.14 | 0.56 | | 0.04 |  | ● |  | ● |
| AWC34_RS00550 | KR domain-containing protein | IQ | 2.65 | 2.11 | | -0.53 |  | ● | ● | ● |
| AWC34_RS12285 | IucA/IucC family siderophore biosynthesis protein | IQ | 3 | 3.33 | | 0.74 |  | ● | ● | ● |
| **Inorganic ion transport and metabolism** | | | | | |  |  |  |  |  |
| AWC34_RS00415 | Catalase | P | 0.96 | 3.42 | | -0.65 |  | ● | ● | ● |
| AWC34_RS00500 | MFS transporter | P | 0.44 | 2.53 | | -1.01 |  | ● | ● | ● |
| AWC34_RS02525 | Inorganic phosphate transporter | P | 0.24 | 2.22 | | -0.86 |  | ● | ● | ● |
| AWC34_RS02735 | HlyC/CorC family transporter | P | 0.63 | 2.24 | | 0.08 |  | ● | ● | ● |
| AWC34_RS03775 | ABC transporter permease | P | 3.05 | 0.95 | | -0.39 |  | ● | ● | ● |
| AWC34_RS03980 | Arsenic transporter | P | -1.06 | 2.15 | | -0.36 |  | ● | ● | ● |
| AWC34_RS05465 | BCCT family transporter | P | 1.71 | 3.69 | | 0.38 |  | ● | ● | ● |
| AWC34_RS05675 | Phosphate ABC transporter, permease protein PstA | P | 0.95 | 2.09 | | 0.49 |  | ● | ● | ● |
| AWC34_RS05685 | Thioredoxin reductase | P | 2.11 | 0.5 | | 1.73 |  | ● | ● | ● |
| AWC34_RS05770 | BCCT family transporter | P | 0.56 | 3.82 | | -0.71 |  | ● | ● | ● |
| AWC34_RS06490 | Metal ABC transporter ATP-binding protein | P | 0.29 | 2.54 | | -0.6 |  | ● | ● | ● |
| AWC34_RS06700 | Divalent metal cation transporter | P | 1.83 | 2.79 | | -0.7 |  | ● | ● | ● |
| AWC34_RS09135 | BCCT family transporter | P | 1.87 | -0.07 | | 2.62 |  | ● | ● | ● |
| AWC34_RS09175 | Energy-coupling factor transporter transmembrane protein EcfT | P | -0.18 | 2.37 | | 0.74 |  | ● | ● | ● |
| AWC34_RS09180 | Energy-coupling factor transporter ATPase | P | 0.31 | 2.39 | | 0.38 |  | ● | ● | ● |
| AWC34_RS09185 | Energy-coupling factor transporter ATPase | P | -0.43 | 2.57 | | 0.46 |  | ● | ● | ● |
| AWC34_RS09595 | QacE family quaternary ammonium compound efflux SMR transporter | P | 0.14 | 2.3 | | -0.23 |  | ● | ● | ● |
| AWC34_RS09670 | Alkaline phosphatase | P | 0.88 | 0.91 | | 2.17 |  | ● | ● | ● |
| AWC34_RS10865 | BCCT family transporter | P | 1.76 | 4.16 | | 0.33 |  | ● | ● | ● |
| AWC34_RS11005 | SulP family inorganic anion transporter | P | -0.44 | 2 | | 0.45 |  | ● | ● | ● |
| AWC34_RS11140 | ABC transporter permease | P | 0.43 | 2.02 | | -0.71 |  | ● | ● | ● |
| AWC34_RS11510 | QacE family quaternary ammonium compound efflux SMR transporter | P | -0.84 | 2.7 | | -0.81 |  | ● | ● | ● |
| AWC34_RS11530 | BCCT family transporter | P | 0.78 | 4.83 | | 0.23 |  | ● | ● | ● |
| **Secondary metabolites biosynthesis, transport and catabolism** | | | | |  | | | | | |
| AWC34_RS09755 | Imidazolonepropionase | Q | 2.24 | 1.87 | | 0.3 |  | ● | ● | ● |
| AWC34_RS12290 | Siderophore biosynthesis protein SbnE | Q | 3.07 | 3.49 | | 0.86 |  | ● | ● | ● |
| AWC34_RS12300 | Siderophore biosynthesis protein SbnC | Q | 3.68 | 3.86 | | 0.89 |  | ● | ● | ● |
| AWC34_RS13320 | D-alanine--poly(phosphoribitol) ligase subunit DltA | Q | 3.95 | 2.71 | | -0.65 |  | ● | ● | ● |
| **General function prediction only** | | | |  | |  |  |  |  |  |
| AWC34_RS00015 | tRNA uridine-5-carboxymethylaminomethyl(34) synthesis GTPase MnmE | R | 1.2 | 2.75 | | 0.66 |  | ● | ● | ● |
| AWC34_RS00305 | Amidohydrolase | R | 3.34 | 2.64 | | 0.4 |  | ● | ● | ● |
| AWC34_RS01850 | Type 1 glutamine amidotransferase | R | 0.78 | -0.65 | | 2.09 |  | ● | ● |  |
| AWC34_RS02230 | Flavodoxin family protein | R | 0.88 | 2.23 | | 0.66 |  | ● | ● | ● |
| AWC34_RS02770 | 7-cyano-7-deazaguanine synthase QueC | R | -0.51 | 3.36 | | 0.78 |  | ● | ● | ● |
| AWC34_RS03140 | Carboxylesterase | R | 0.92 | 5.01 | | 0.23 |  | ● | ● | ● |
| AWC34_RS05290 | GTPase HflX | R | 1.31 | 2.01 | | 2.22 |  | ● | ● | ● |
| AWC34_RS05565 | 4-oxalocrotonate tautomerase | R | -2.21 | -1.86 | | 2.12 |  | ● | ● | ● |
| AWC34_RS06050 | Hypothetical protein | R | -1.79 | 0 | | 2.06 |  | ● | ● | ● |
| AWC34_RS06705 | LamB/YcsF family protein | R | 2.57 | 3.03 | | 0.52 |  | ● | ● | ● |
| AWC34_RS06795 | ABC transporter ATP-binding protein | R | -1.31 | 0.08 | | 5.17 |  | ● | ● | ● |
| AWC34_RS08360 | Nitroreductase | R | -0.42 | 2.41 | | -0.42 |  | ● | ● | ● |
| AWC34_RS09155 | Alpha/beta hydrolase | R | 1.11 | 0.68 | | 2.03 |  | ● | ● | ● |
| AWC34_RS09940 | N-acetyltransferase | R | -1.4 | -2.43 | | 3.12 |  | ● | ● | ● |
| AWC34_RS09945 | Quinone oxidoreductase | R | 1.09 | 3.53 | | -0.48 |  | ● | ● | ● |
| AWC34_RS10335 | Thioesterase | R | -0.26 | 4.49 | | -0.72 |  | ● | ● | ● |
| AWC34_RS10910 | MMPL family transporter | R | 0.89 | 2.14 | | -0.72 |  | ● | ● | ● |
| AWC34_RS10990 | KR domain-containing protein | R | 0.92 | -0.3 | | 2.08 |  | ● | ● | ● |
| AWC34_RS11100 | HAD family hydrolase | R | 0.12 | 3.16 | | 0.85 |  | ● | ● | ● |
| AWC34_RS11105 | Glyoxalase | R | -0.37 | 3.14 | | 0.52 |  | ● | ● | ● |
| AWC34_RS11115 | Msr family ABC-F type ribosomal protection protein | R | -0.38 | 4.88 | | 0.33 |  | ● |  |  |
| AWC34_RS12055 | Rhodanese domain-containing protein | R | 0.4 | 2.03 | | -0.22 |  | ● | ● | ● |
| **Function unknown** | |  |  |  | |  |  |  |  |  |
| AWC34_RS00385 | Hypothetical protein | S | 0.56 | -0.52 | | 2.28 |  | ● | ● | ● |
| AWC34_RS00665 | Hypothetical protein | S | 0.79 | 3.85 | | 1.41 |  | ● | ● | ● |
| AWC34_RS00915 | Hypothetical protein | S | -0.91 | 2.52 | | -0.18 |  | ● | ● | ● |
| AWC34_RS01720 | Hypothetical protein | S | -0.66 | 3.09 | | -0.56 |  | ● |  | ● |
| AWC34_RS02490 | Membrane protein | S | 1.16 | 3.75 | | -0.79 |  | ● | ● | ● |
| AWC34_RS03990 | GlsB/YeaQ/YmgE family stress response membrane protein | S | 0.9 | 2.64 | | 0.43 |  | ● | ● | ● |
| AWC34_RS04415 | DNA-binding protein | S | -0.11 | 4.54 | | 0.39 |  | ● | ● | ● |
| AWC34_RS05940 | Membrane protein | S | -1.04 | 2.75 | | 0.55 |  | ● | ● | ● |
| AWC34_RS06270 | BrxA/BrxB family bacilliredoxin | S | -0.44 | -0.72 | | 2.33 |  | ● | ● | ● |
| AWC34_RS07625 | Peptidase | S | 0.56 | 0.98 | | 2.29 |  | ● | ● | ● |
| AWC34_RS07750 | DUF445 domain-containing protein | S | 0.44 | 2.2 | | -1.09 |  | ● | ● | ● |
| AWC34_RS08410 | YeeE/YedE family protein | S | 0.7 | 3.04 | | 0.05 |  | ● | ● | ● |
| AWC34_RS08650 | Transglycosylase | S | 3.04 | 4.54 | | 2.97 |  | ● | ● | ● |
| AWC34_RS09560 | CHAP domain-containing protein | S | 0.74 | 3.5 | | 0.67 |  | ● | ● | ● |
| AWC34_RS09585 | CHAP domain-containing protein | S | 0.21 | 4.27 | | 1.8 |  | ● | ● | ● |
| AWC34_RS09590 | QacE family quaternary ammonium compound efflux SMR transporter | S | -0.49 | 2.12 | | 0.64 |  | ● | ● | ● |
| AWC34_RS09765 | Histidine transporter | S | 2.06 | 1.02 | | -0.59 |  | ● | ● | ● |
| AWC34_RS10220 | Hypothetical protein | S | 2.12 | 3.05 | | 2.52 |  | ● | ● | ● |
| AWC34_RS10345 | 3-keto-5-aminohexanoate cleavage protein | S | -0.51 | 2.7 | | 0.31 |  | ● | ● | ● |
| AWC34_RS10575 | Antibiotic biosynthesis monooxygenase | S | -1.35 | -0.99 | | 2.53 |  | ● | ● | ● |
| AWC34_RS10595 | Membrane protein | S | 0.06 | 3 | | 0.55 |  | ● | ● | ● |
| AWC34_RS11465 | Hypothetical protein | S | 2.24 | 1.18 | | 0.37 |  | ● | ● | ● |
| AWC34_RS11790 | Hypothetical protein | S | 0.88 | 2.2 | | 0.42 |  | ● |  |  |
| AWC34_RS12070 | Transglycosylase | S | 0.81 | 3.44 | | 4.41 |  | ● | ● | ● |
| AWC34_RS12080 | Transglycosylase | S | 2.12 | 4.85 | | 3.52 |  | ● | ● |  |
| AWC34_RS12140 | YitT family protein | S | -2.25 | 3.59 | | -0.52 |  | ● | ● | ● |
| AWC34_RS12375 | Tautomerase family protein | S | 0.22 | -1.33 | | 2.13 |  | ● | ● | ● |
| AWC34_RS12420 | Hypothetical protein | S | 0.21 | 2.21 | | 0.32 |  | ● | ● | ● |
| AWC34_RS12440 | DUF805 domain-containing protein | S | 2.89 | 1.82 | | -0.12 |  | ● |  |  |
| AWC34_RS02530 | LysM peptidoglycan-binding domain-containing protein | SM | 1.4 | 2.55 | | 0.74 |  | ● | ● | ● |
| AWC34_RS03000 | CHAP domain-containing protein | SM | 1.77 | 2.41 | | 4.03 |  | ● | ● | ● |
| AWC34_RS11750 | M23 family peptidase | SM | -0.28 | 2.92 | | 3.82 |  | ● | ● | ● |

**Supplementary Table 7.** Summary of genes down-regulated by chloramphenicol, erythromycin and lincomycin.

| **Gene locus** | **Product** | **COG** | **Log2 (Fold change)** | | |  | **Comparative** | | |
| --- | --- | --- | --- | --- | --- | --- | --- | --- | --- |
|  |  |  | **CHL** | **ERY** | **LIN** |  | **KM1031** | **C2014** | **KS1039** |
| **Translation, ribosomal structure and biogenesis** | | | | | |  |  |  |  |
| AWC34_RS03620 | General stress protein | J | -2.13 | -1.04 | -0.25 |  | ● | ● | ● |
| AWC34_RS03645 | N-acetyltransferase | J | -2.67 | -2.94 | -1.28 |  | ● | ● | ● |
| AWC34_RS07270 | GNAT family N-acetyltransferase | J | -2.24 | -3.91 | -0.56 |  | ● | ● | ● |
| AWC34_RS10795 | N-acetyltransferase | J | -1.4 | -2.48 | -0.15 |  | ● | ● | ● |
| **Transcription, regulators** | | | | | |  |  |  |  |
| AWC34_RS00430 | MarR family transcriptional regulator | K | -1.41 | -2.19 | 0.4 |  | ● | ● | ● |
| AWC34_RS00880 | RNA polymerase sigma factor | K | -1 | -2.24 | 0.65 |  | ● | ● | ● |
| AWC34_RS01355 | CtsR family transcriptional regulator | K | -2.14 | -1.67 | 0.56 |  | ● | ● | ● |
| AWC34_RS01665 | LacI family DNA-binding transcriptional regulator | K | -1.94 | -2.07 | -1.07 |  | ● | ● | ● |
| AWC34_RS01785 | Lrp/AsnC family transcriptional regulator | K | -2 | -0.61 | -1.09 |  | ● |  | ● |
| AWC34_RS02060 | Transcriptional regulator | K | -2.42 | -1.88 | -2.23 |  | ● | ● | ● |
| AWC34_RS02305 | Transcriptional regulator | K | -2.12 | -3.15 | -1.94 |  | ● | ● | ● |
| AWC34_RS03230 | Cold-shock protein | K | -2.2 | 2.91 | 0.93 |  | ● | ● | ● |
| AWC34_RS03975 | ArsR family transcriptional regulator | K | -2.19 | 0.87 | -0.27 |  |  |  |  |
| AWC34_RS04030 | MarR family transcriptional regulator | K | -0.53 | 0.31 | -2.32 |  | ● | ● | ● |
| AWC34_RS04045 | MarR family transcriptional regulator | K | -4.16 | -2.54 | 0.12 |  | ● | ● | ● |
| AWC34_RS05505 | Transcription antiterminator | K | -3.25 | -3.75 | -0.95 |  | ● | ● | ● |
| AWC34_RS07470 | Transcriptional regulator | K | -0.93 | -2.31 | -0.71 |  | ● | ● | ● |
| AWC34_RS07530 | Sigma-70 family RNA polymerase sigma factor | K | -1.53 | -3.1 | -1.33 |  | ● | ● | ● |
| AWC34_RS08315 | GntR family transcriptional regulator | K | -2.36 | 0.67 | 0.73 |  | ● | ● | ● |
| AWC34_RS08400 | LacI family transcriptional regulator | K | -2.19 | -1.47 | -0.41 |  | ● | ● | ● |
| AWC34_RS09545 | Spermidine/putrescine ABC transporter ATP-binding protein | K | -2.82 | -5.04 | -0.66 |  | ● | ● | ● |
| AWC34_RS09720 | MurR/RpiR family transcriptional regulator | K | -2.24 | -0.49 | -0.67 |  | ● | ● | ● |
| AWC34_RS09885 | MarR family transcriptional regulator | K | -0.75 | -2.16 | 0.82 |  | ● | ● | ● |
| AWC34_RS10050 | AraC family transcriptional regulator | K | -2.08 | -1.87 | -0.69 |  | ● |  | ● |
| AWC34_RS10080 | DNA-binding response regulator | K | -2.64 | -1.03 | -1.11 |  | ● | ● | ● |
| AWC34_RS10165 | PadR family transcriptional regulator | K | -1.48 | -3.01 | -1.12 |  | ● | ● | ● |
| AWC34_RS10770 | N-acetyltransferase | K | -2.17 | -3.06 | -0.96 |  | ● | ● | ● |
| AWC34_RS11505 | N-acetyltransferase | K | -0.18 | -2.35 | 1.2 |  | ● | ● | ● |
| AWC34_RS11625 | Transcriptional regulator | K | -1.37 | -2.39 | 0.36 |  | ● | ● | ● |
| AWC34_RS11720 | MarR family transcriptional regulator | K | -1.14 | -2.37 | 0.28 |  | ● | ● | ● |
| AWC34_RS12355 | LysR family transcriptional regulator | K | -0.91 | -2.3 | -1.43 |  | ● | ● | ● |
| AWC34_RS12370 | AraC family transcriptional regulator | K | -1.95 | -3.33 | -1.61 |  | ● | ● | ● |
| AWC34_RS12400 | MerR family DNA-binding transcriptional regulator | K | -4.36 | -5.44 | 0.17 |  | ● | ● | ● |
| AWC34_RS12885 | Transcriptional regulator | K | -2.73 | -1.68 | -1.4 |  | ● |  |  |
| AWC34_RS12920 | ArsR family transcriptional regulator | K | -1.95 | -1.04 | -2.23 |  | ● | ● | ● |
| AWC34_RS12945 | ArsR family transcriptional regulator | K | -2.44 | -1.3 | -2.4 |  | ● | ● | ● |
| AWC34_RS13125 | Transcriptional regulator | K | -3.85 | -1.69 | -1.22 |  |  |  |  |
| AWC34_RS13255 | Transcriptional regulator | K | -3.85 | -1.69 | -1.22 |  |  |  |  |
| AWC34_RS13165 | Betaine-aldehyde dehydrogenase | KC | -1.64 | -2.65 | -0.86 |  | ● |  |  |
| AWC34_RS10905 | Hypothetical protein | KG | -3.9 | -4.63 | 0.17 |  | ● |  | ● |
| **Replication, recombination and repair** | | | | | |  |  |  |  |
| AWC34_RS01105 | GIY-YIG nuclease family protein | L | -1.7 | -2.42 | 0.06 |  | ● | ● | ● |
| AWC34_RS02335 | DNA integrase | L | -2.93 | -0.71 | 0.37 |  | ● | ● | ● |
| AWC34_RS03495 | IS1182 family transposase | L | -0.49 | -2.85 | -0.67 |  | ● | ● |  |
| AWC34_RS04950 | DNA-protecting protein DprA | L | -2.36 | -0.8 | -0.74 |  | ● | ● | ● |
| AWC34_RS12255 | (deoxy)nucleoside triphosphate pyrophosphohydrolase | L | -1.99 | -3.97 | 0.04 |  | ● | ● | ● |
| AWC34_RS12845 | Recombinase family protein | L | -2.18 | -0.54 | 0.17 |  | ● | ● |  |
| AWC34_RS13025 | Recombinase family protein | L | -2.18 | -0.54 | 0.17 |  | ● |  |  |
| AWC34_RS13295 | Protein rep | L | -2.6 | -0.86 | -1.1 |  | ● |  |  |
| AWC34_RS13445 | Hypothetical protein | L | -0.72 | -6.19 | -1.26 |  | ● |  |  |
| **Cell cycle control, cell division, chromosome partitioning** | | | | | |  |  |  |  |
| AWC34_RS04580 | Cell division protein FtsL | D | -2.18 | 0.57 | 0.86 |  | ● | ● | ● |
| **Cell wall/membrane/envelope biogenesis** | | | | | |  |  |  |  |
| AWC34_RS00055 | Hypothetical protein | M | -2.69 | -1.97 | 0.62 |  | ● |  |  |
| AWC34_RS01535 | UDP-glucose 4-epimerase | M | -0.13 | -2.01 | 0.16 |  | ● |  |  |
| AWC34_RS01545 | UDP-N-acetylglucosamine 2-epimerase (non-hydrolyzing) | M | 0.92 | -2.09 | 0.12 |  | ● |  |  |
| AWC34_RS01555 | Capsular biosynthesis protein | M | -0.29 | -2.59 | 0.07 |  | ● |  |  |
| AWC34_RS01565 | Capsular biosynthesis protein | M | -1.68 | -2.31 | -1.33 |  | ● |  |  |
| AWC34_RS01570 | Nucleotide sugar dehydrogenase | M | -0.07 | -2.14 | -0.51 |  | ● |  |  |
| AWC34_RS01585 | Hypothetical protein | M | -0.75 | -2.84 | -1.01 |  | ● |  |  |
| AWC34_RS03510 | D-alanyl-lipoteichoic acid biosynthesis protein DltB | M | -0.7 | -3.64 | -2.65 |  | ● | ● | ● |
| AWC34_RS03520 | D-alanyl-lipoteichoic acid biosynthesis protein DltD | M | -1.79 | -2.93 | -1.58 |  | ● | ● | ● |
| AWC34_RS04425 | M23 family peptidase | M | -2.73 | -0.04 | 0.05 |  | ● | ● | ● |
| AWC34_RS04575 | 16S rRNA (cytosine(1402)-N(4))-methyltransferase RsmH | M | -3.8 | -1.17 | 0.28 |  | ● | ● | ● |
| AWC34_RS07615 | Glycosyltransferase family 2 protein | M | -3.85 | -4.93 | -0.04 |  | ● |  | ● |
| AWC34_RS12225 | Glycosyltransferase | M | -1.52 | -2.11 | -1.64 |  | ● |  | ● |
| **Post-translational modification, protein turnover, chaperones** | | | | | |  |  |  |  |
| AWC34_RS03740 | ATP-dependent chaperone ClpB | O | -2 | -0.99 | 0.13 |  | ● | ● | ● |
| AWC34_RS05445 | DUF1453 domain-containing protein | O | -2.55 | -1.75 | -1.44 |  | ● | ● | ● |
| AWC34_RS07990 | Peroxiredoxin | O | -0.38 | -2.99 | 1.37 |  | ● | ● | ● |
| AWC34_RS08345 | Co-chaperone GroES | O | -1.39 | -2.54 | -0.08 |  | ● | ● | ● |
| AWC34_RS11770 | Hypothetical protein | O | -2.41 | -1.75 | 0.96 |  | ● | ● |  |
| AWC34_RS11880 | Anaerobic ribonucleoside-triphosphate reductase activating protein | O | -2.08 | -0.87 | 1.52 |  | ● | ● | ● |
| **Signal transduction mechanisms** | | | | | |  |  |  |  |
| AWC34_RS05280 | RNA chaperone Hfq | T | -1.67 | -2.01 | -1.94 |  | ● | ● | ● |
| AWC34_RS08370 | Accessory gene regulator B | T | 0.13 | -2.2 | -2.38 |  | ● | ● | ● |
| AWC34_RS08380 | GHKL domain-containing protein | T | -0.84 | -1.17 | -2.96 |  | ● | ● | ● |
| AWC34_RS08385 | DNA-binding response regulator | T | -2.48 | -0.56 | -1.1 |  | ● | ● | ● |
| AWC34_RS08550 | Type II toxin-antitoxin system PemK/MazF family toxin | T | -2.03 | -0.64 | 0.9 |  | ● | ● | ● |
| AWC34_RS10085 | Sensor histidine kinase | T | -2.4 | -1.42 | -0.96 |  | ● | ● | ● |
| **Intracellular trafficking, secretion, and vesicular transport** | | | | | |  |  |  |  |
| AWC34_RS04885 | Signal recognition particle protein | U | 0.63 | 0.87 | -2.06 |  | ● | ● | ● |
| AWC34_RS06405 | Competence protein | U | -2.55 | -4.95 | -0.69 |  | ● | ● | ● |
| AWC34_RS06425 | Type II secretion system F family protein | U | -2.92 | -1.72 | -0.32 |  | ● | ● | ● |
| AWC34_RS06430 | Competence protein ComGA | U | -2.41 | -0.94 | 0.28 |  | ● | ● | ● |
| **Energy production and conversion** | | | | | |  |  |  |  |
| AWC34_RS00575 | NADH dehydrogenase subunit 5 | C | -3.18 | -1.66 | -1.02 |  | ● | ● | ● |
| AWC34_RS04085 | Cytochrome aa3 quinol oxidase subunit IV | C | -0.85 | -2.26 | -1.84 |  | ● | ● | ● |
| AWC34_RS04480 | Succinate dehydrogenase | C | -0.42 | -2.19 | -0.62 |  | ● | ● | ● |
| AWC34_RS04525 | Hypothetical protein | C | -2.08 | -2.75 | 0.24 |  | ● | ● | ● |
| AWC34_RS06295 | Butyrate kinase | C | -0.93 | -2.11 | -0.85 |  | ● | ● | ● |
| AWC34_RS06300 | Phosphate butyryltransferase | C | -0.59 | -2.27 | -0.53 |  | ● | ● | ● |
| AWC34_RS07275 | Glycerophosphoryl diester phosphodiesterase | C | -1.62 | -2.21 | -0.26 |  | ● | ● | ● |
| AWC34_RS08720 | ATP synthase | C | -5.55 | -4.48 | -1.49 |  | ● | ● | ● |
| AWC34_RS10095 | Respiratory nitrate reductase subunit gamma | C | -3.59 | -2.34 | -1.19 |  | ● | ● | ● |
| AWC34_RS10100 | Nitrate reductase molybdenum cofactor assembly chaperone | C | -5.8 | -3.52 | -1.4 |  | ● | ● | ● |
| AWC34_RS10105 | Nitrate reductase subunit beta | C | -5.2 | -2.19 | -1.1 |  | ● | ● | ● |
| AWC34_RS10110 | Nitrate reductase subunit alpha | C | -4.22 | -2.02 | -0.88 |  | ● | ● | ● |
| AWC34_RS10120 | Nitrite reductase (NAD(P)H) small subunit | C | -4.29 | -3.5 | -0.82 |  | ● | ● | ● |
| AWC34_RS10125 | Nitrite reductase large subunit | C | -3.84 | -2.59 | -0.89 |  | ● | ● | ● |
| **Amino acid transport and metabolism** | | | | | |  |  |  |  |
| AWC34_RS06260 | Hypothetical protein | E | -2.21 | 0.72 | -0.1 |  | ● | ● | ● |
| AWC34_RS07485 | Proline dehydrogenase | E | -0.71 | -2.56 | -1.1 |  | ● | ● | ● |
| AWC34_RS08230 | Prephenate dehydratase | E | -2.95 | -1.25 | 0.24 |  | ● | ● | ● |
| AWC34_RS08225 | Nitric oxide synthase | EP | -2.23 | -1.49 | -0.27 |  | ● | ● | ● |
| **Nucleotide transport and metabolism** | | | | | |  |  |  |  |
| AWC34_RS03185 | ATPase AAA | F | -1.87 | -2.39 | -0.5 |  | ● | ● | ● |
| AWC34_RS06090 | Nucleoside-diphosphate kinase | F | -1.1 | -2.25 | 0.42 |  | ● | ● | ● |
| **Carbohydrate transport and metabolism** | | | | | |  |  |  |  |
| AWC34_RS00640 | PTS sugar transporter subunit IIB | G | -1.16 | -2.47 | -0.45 |  | ● | ● | ● |
| AWC34_RS00650 | PTS lactose/cellobiose transporter subunit IIA | G | -2.63 | -3.3 | -0.58 |  | ● | ● | ● |
| AWC34_RS01660 | Hypothetical protein | G | -3.09 | -0.64 | -1.06 |  | ● |  |  |
| AWC34_RS02205 | Trehalose utilization protein | G | 0.65 | -2.03 | -0.06 |  | ● | ● | ● |
| AWC34_RS05240 | Aquaporin family protein | G | -1.32 | -2.3 | -0.35 |  | ● | ● | ● |
| AWC34_RS05535 | D-ribose pyranase | G | -0.86 | -4.41 | 0.2 |  | ● | ● | ● |
| AWC34_RS09990 | Dihydroxyacetone kinase subunit DhaK | G | -1.4 | -2.11 | -0.26 |  | ● | ● | ● |
| AWC34_RS10510 | Beta-glucuronidase | G | -2.13 | -0.76 | -0.64 |  | ● |  | ● |
| AWC34_RS10515 | MFS transporter | G | -3.07 | -2.2 | -0.11 |  | ● |  | ● |
| AWC34_RS10900 | MFS transporter | G | -2.23 | -0.71 | 0.13 |  | ● |  | ● |
| AWC34_RS11310 | MFS transporter | G | -2.61 | -0.29 | -0.43 |  | ● |  | ● |
| AWC34_RS12035 | D-ribose pyranase | G | -2.07 | -2.15 | 0.1 |  | ● | ● | ● |
| AWC34_RS00145 | PRD domain-containing protein | GK | -1.25 | -2.81 | -0.84 |  | ● |  |  |
| **Coenzyme transport and metabolism** | | | | | |  |  |  |  |
| AWC34_RS10115 | Uroporphyrinogen-III C-methyltransferase | H | -4.46 | -3.46 | -1.06 |  | ● | ● | ● |
| AWC34_RS10130 | Bifunctional precorrin-2 dehydrogenase/sirohydrochlorin ferrochelatase | H | -3.21 | -2.72 | -0.47 |  | ● | ● | ● |
| AWC34_RS10135 | Hypothetical protein | H | -3.61 | -3.54 | -1.34 |  | ● | ● | ● |
| AWC34_RS11715 | 2-dehydropantoate 2-reductase | H | -1.18 | -2.13 | -0.05 |  | ● | ● | ● |
| **Lipid transport and metabolism** | | | | | |  |  |  |  |
| AWC34_RS00440 | Hypothetical protein | I | -2.54 | -2.33 | -0.16 |  | ● | ● | ● |
| AWC34_RS03515 | D-alanine--poly(phosphoribitol) ligase subunit 2 | I | -0.75 | -3.41 | -1.54 |  | ● | ● | ● |
| AWC34_RS03730 | Acetyltransferase | I | -2.24 | -3.11 | -0.62 |  | ● |  |  |
| AWC34_RS07480 | Alpha/beta hydrolase | I | -2.38 | -1.19 | 1.68 |  | ● | ● | ● |
| **Inorganic ion transport and metabolism** | | | | | |  |  |  |  |
| AWC34_RS00695 | QacE family quaternary ammonium compound efflux SMR transporter | P | -4.1 | -2.18 | -1.35 |  | ● | ● | ● |
| AWC34_RS00935 | Sodium-dependent transporter | P | -1.66 | -0.69 | -2.31 |  | ● | ● | ● |
| AWC34_RS03365 | Arsenate reductase family protein | P | -0.67 | -3.08 | 0.97 |  | ● | ● | ● |
| AWC34_RS03885 | Esterase family protein | P | -2.33 | -0.8 | -0.3 |  | ● | ● | ● |
| AWC34_RS05540 | Sugar ABC transporter ATP-binding protein | P | -0.87 | -3.35 | 0.03 |  | ● | ● | ● |
| AWC34_RS07445 | Rhodanese-like domain-containing protein | P | 0.68 | -2.18 | 1.47 |  | ● | ● | ● |
| AWC34_RS08940 | ABC transporter ATP-binding protein | P | -0.05 | -2.33 | 0.33 |  | ● | ● | ● |
| AWC34_RS09835 | Magnesium and cobalt transport protein CorA | P | -2.39 | -3.49 | -0.52 |  | ● | ● | ● |
| AWC34_RS10075 | NarK/NasA family nitrate transporter | P | -3.84 | -2.31 | -0.58 |  | ● | ● | ● |
| AWC34_RS10985 | Bacteriocin ABC transporter ATP-binding protein | P | -2.91 | -3.38 | 0.03 |  | ● |  | ● |
| AWC34_RS12090 | Membrane protein | P | -2.15 | -1.2 | -0.25 |  | ● |  | ● |
| **Secondary metabolites biosynthesis, transport and catabolism** | | | | | |  |  |  |  |
| AWC34_RS04840 | Transcription factor FapR | Q | -4.44 | -4.33 | -1.43 |  | ● | ● | ● |
| AWC34_RS12165 | TRAP transporter small permease | Q | -2.11 | -2.46 | 0.25 |  | ● |  |  |
| **General function prediction only** | | | | | |  |  |  |  |
| AWC34_RS01950 | Pyridoxamine 5'-phosphate oxidase | R | -0.8 | -3.41 | 0.59 |  | ● | ● | ● |
| AWC34_RS04455 | CvpA family protein | R | -1.82 | -2.7 | -1.4 |  | ● | ● | ● |
| AWC34_RS04535 | N-acetyltransferase | R | -0.25 | -2.98 | -1.46 |  | ● | ● | ● |
| AWC34_RS05565 | 4-oxalocrotonate tautomerase | R | -2.21 | -1.86 | 2.12 |  | ● | ● | ● |
| AWC34_RS05700 | Aldo/keto reductase | R | -0.94 | -2.25 | 0.54 |  | ● | ● | ● |
| AWC34_RS05805 | ABC transporter ATP-binding protein | R | -1.62 | -2.42 | -1.39 |  | ● |  | ● |
| AWC34_RS06640 | DNA internalization-related competence protein ComEC/Rec2 | R | -2.59 | -1.46 | -1.21 |  | ● | ● | ● |
| AWC34_RS08040 | Recombination regulator RecX | R | 0.4 | -2.72 | -0.41 |  | ● | ● | ● |
| AWC34_RS08350 | CPBP family intramembrane metalloprotease | R | -1.17 | 0.76 | -2.1 |  | ● | ● | ● |
| AWC34_RS08815 | VOC family protein | R | -4.08 | -3.9 | -0.34 |  | ● | ● | ● |
| AWC34_RS09940 | N-acetyltransferase | R | -1.4 | -2.43 | 3.12 |  | ● | ● | ● |
| AWC34_RS10205 | Lantibiotic ABC transporter ATP-binding protein | R | -1.11 | -2.02 | -1.56 |  | ● | ● | ● |
| AWC34_RS10210 | ABC transporter permease | R | -1.2 | -3.91 | -3.95 |  | ● | ● | ● |
| AWC34_RS11200 | Thioesterase | R | -4.22 | -2.69 | -0.76 |  | ● | ● | ● |
| AWC34_RS11405 | Hypothetical protein | R | -0.86 | -2.08 | -1.23 |  | ● | ● | ● |
| AWC34_RS11845 | Pyridoxamine 5'-phosphate oxidase | R | -1.03 | -3.21 | 1.97 |  | ● | ● | ● |
| AWC34_RS12060 | HAD family hydrolase | R | -2.35 | -2.41 | -0.46 |  | ● | ● | ● |
| AWC34_RS13370 | ABC transporter permease | R | -1.39 | -2.34 | 0.78 |  | ● | ● | ● |
| **Function unknown** | | | | | |  |  |  |  |
| AWC34_RS00100 | Stage II sporulation protein M | S | -1.1 | -2.84 | -0.48 |  | ● |  |  |
| AWC34_RS00460 | Hypothetical protein | S | -2.37 | -2.04 | -0.37 |  | ● |  | ● |
| AWC34_RS00465 | Hypothetical protein | S | -2.44 | -1.6 | -0.89 |  | ● |  | ● |
| AWC34_RS00525 | NINE protein | S | 0.55 | -3.37 | -0.43 |  | ● | ● | ● |
| AWC34_RS00580 | DUF2309 domain-containing protein | S | -3.03 | -1.5 | -1.66 |  | ● | ● | ● |
| AWC34_RS00690 | Multidrug resistance protein SMR | S | -4.9 | -1.28 | -1.65 |  | ● | ● | ● |
| AWC34_RS00855 | Hypothetical protein | S | -3.05 | 0.18 | -1.69 |  | ● | ● | ● |
| AWC34_RS01755 | Hypothetical protein | S | -1.1 | -2.53 | 1.41 |  | ● | ● | ● |
| AWC34_RS01885 | Metal-sensitive transcriptional regulator | S | -3.18 | -2.78 | 0.74 |  | ● |  |  |
| AWC34_RS01890 | Sulfite exporter TauE/SafE family protein | S | -3.39 | -0.62 | -0.4 |  | ● |  |  |
| AWC34_RS01910 | Hypothetical protein | S | -2.45 | -1.88 | 0.5 |  | ● | ● | ● |
| AWC34_RS01925 | Hypothetical protein | S | 0.38 | -2.23 | -0.17 |  | ● | ● |  |
| AWC34_RS02585 | DUF1129 domain-containing protein | S | -2.63 | -1.77 | -0.96 |  | ● | ● | ● |
| AWC34_RS02840 | 5'-3'-deoxyribonucleotidase | S | -2.01 | -1.72 | 0.62 |  | ● | ● | ● |
| AWC34_RS03170 | DUF418 domain-containing protein | S | -2.02 | -2.17 | -1.94 |  | ● | ● | ● |
| AWC34_RS03220 | DUF1648 domain-containing protein | S | -2.04 | -2.64 | -1.09 |  | ● | ● | ● |
| AWC34_RS03315 | NINE protein | S | -0.84 | -3.93 | 0.1 |  | ● | ● | ● |
| AWC34_RS03410 | DUF368 domain-containing protein | S | -0.46 | 0.49 | -2.24 |  | ● | ● | ● |
| AWC34_RS03475 | DUF3055 domain-containing protein | S | -2.17 | -1.03 | -0.54 |  | ● | ● | ● |
| AWC34_RS03480 | DUF86 domain-containing protein | S | -1.12 | -2.43 | -0.01 |  | ● | ● | ● |
| AWC34_RS03650 | DUF454 domain-containing protein | S | -1.88 | -0.78 | -2.03 |  | ● | ● | ● |
| AWC34_RS03725 | Metal-sulfur cluster assembly factor | S | -1.83 | -2.14 | 0.75 |  | ● | ● | ● |
| AWC34_RS03995 | NINE protein | S | -1.29 | -4.21 | 1.08 |  | ● | ● | ● |
| AWC34_RS04240 | Hypothetical protein | S | -1.28 | -2.56 | 0.51 |  | ● | ● | ● |
| AWC34_RS04315 | DUF1054 domain-containing protein | S | -1.54 | -2.45 | 0.21 |  | ● | ● | ● |
| AWC34_RS04340 | Hypothetical protein | S | -3.09 | -1.78 | 0.05 |  | ● | ● | ● |
| AWC34_RS04375 | Hypothetical protein | S | -2.03 | -0.7 | -0.08 |  | ● | ● | ● |
| AWC34_RS04390 | DUF2129 domain-containing protein | S | -2.01 | -0.53 | 1.35 |  | ● | ● | ● |
| AWC34_RS04450 | Cell division protein ZapA | S | -3.18 | -1.56 | -0.05 |  | ● | ● | ● |
| AWC34_RS04570 | Transcriptional regulator MraZ | S | -3.39 | -0.81 | -0.29 |  | ● | ● | ● |
| AWC34_RS04630 | YggT family protein | S | -0.9 | 0.32 | -2.12 |  | ● | ● | ● |
| AWC34_RS04880 | Putative DNA-binding protein | S | 0.73 | -1.14 | -2.36 |  | ● | ● | ● |
| AWC34_RS05760 | 5-bromo-4-chloroindolyl phosphate hydrolysis protein | S | -1.2 | -2.06 | 0.82 |  | ● | ● | ● |
| AWC34_RS05885 | YozE family protein | S | -0.27 | -3.02 | -0.2 |  | ● | ● | ● |
| AWC34_RS05905 | DegV family protein | S | 0.56 | -2.03 | 0.08 |  | ● | ● | ● |
| AWC34_RS06440 | Hypothetical protein | S | -0.42 | -2.26 | -0.19 |  | ● | ● | ● |
| AWC34_RS06450 | DUF910 domain-containing protein | S | -0.63 | -0.71 | -2.97 |  | ● | ● | ● |
| AWC34_RS07395 | Hypothetical protein | S | -3.23 | -1.9 | -0.93 |  | ● | ● | ● |
| AWC34_RS07545 | Pentapeptide repeat-containing protein | S | -1.34 | -2.43 | -1.57 |  | ● | ● | ● |
| AWC34_RS08035 | DUF1811 domain-containing protein | S | -0.23 | -3.8 | -0.3 |  | ● | ● | ● |
| AWC34_RS08675 | DUF1146 domain-containing protein | S | -1.27 | -2.81 | 0.67 |  | ● | ● | ● |
| AWC34_RS08845 | Hypothetical protein | S | 0.23 | -2.23 | -2.46 |  | ● | ● | ● |
| AWC34_RS08880 | DUF393 domain-containing protein | S | -0.51 | -2.11 | 0.14 |  | ● | ● | ● |
| AWC34_RS08945 | DUF2200 domain-containing protein | S | -0.25 | -2.1 | 1.15 |  | ● |  | ● |
| AWC34_RS09075 | Hypothetical protein | S | -1.52 | -5.3 | -2.22 |  | ● | ● | ● |
| AWC34_RS09335 | Hypothetical protein | S | -1.07 | -2.91 | -1.48 |  | ● | ● | ● |
| AWC34_RS09580 | DUF4870 domain-containing protein | S | -1.06 | -2.57 | 0.45 |  | ● | ● | ● |
| AWC34_RS10090 | GAF domain-containing protein | S | -2.72 | -1.05 | -1.29 |  | ● | ● | ● |
| AWC34_RS10170 | Hypothetical protein | S | -0.5 | -2.94 | -1.61 |  | ● | ● | ● |
| AWC34_RS10175 | Polyisoprenoid-binding protein | S | 0.17 | -2.1 | 0.71 |  | ● | ● | ● |
| AWC34_RS10230 | GtrA family protein | S | -0.91 | -3.63 | -0.94 |  |  |  |  |
| AWC34_RS10270 | DUF969 domain-containing protein | S | -0.47 | -2.52 | -0.88 |  | ● | ● | ● |
| AWC34_RS10545 | GTP pyrophosphokinase | S | -1.4 | -2.22 | 1.47 |  | ● | ● | ● |
| AWC34_RS10875 | Hypothetical protein | S | -1.41 | -3.15 | -0.86 |  | ● | ● | ● |
| AWC34_RS10975 | DUF1430 domain-containing protein | S | -2.37 | -2.14 | -1.02 |  | ● |  | ● |
| AWC34_RS10980 | Hypothetical protein | S | -2.21 | -2.6 | -0.16 |  | ● |  | ● |
| AWC34_RS11080 | DUF896 family protein | S | -1.27 | -2 | -0.29 |  | ● | ● | ● |
| AWC34_RS11270 | Tripartite tricarboxylate transporter TctB family protein | S | -2.14 | -3.07 | -2.12 |  | ● | ● | ● |
| AWC34_RS11315 | Hypothetical protein | S | -0.91 | -2.2 | -0.2 |  | ● |  | ● |
| AWC34_RS12140 | YitT family protein | S | -2.25 | 3.59 | -0.52 |  | ● | ● | ● |
| AWC34_RS12220 | Hypothetical protein | S | -1.71 | -3.07 | -1.58 |  | ● |  | ● |
| AWC34_RS12615 | DUF1643 domain-containing protein | S | -3.21 | -0.05 | -0.85 |  | ● |  | ● |
| AWC34_RS12620 | Hypothetical protein | S | -4.11 | -2.97 | -0.2 |  | ● |  | ● |
| AWC34_RS12665 | DUF2357 domain-containing protein | S | -0.93 | -2.09 | -1.03 |  | ● |  |  |
| AWC34_RS13305 | FeoB-associated Cys-rich membrane protein | S | - | -1.14 | -4.39 |  | ● | ● | ● |

**Supplementary Table 8**. Significantly different expressed genes related with efflux, transporter and salt tolerance by chloramphenicol, erythromycin and lincomycin.

| **Gene** | **Gene locus** | **Product** | **Log2 (Fold change)** | | | **COG** |
| --- | --- | --- | --- | --- | --- | --- |
|  |  |  | **CHL** | **ERY** | **LIN** |  |
| **Efflux** |  |  |  |  |  |  |
| *cynX* | AWC34_RS11585 | MFS transporter | -0.56 | 2.06 | -0.02 | K |
| *emrB* | AWC34_RS10200 | MFS transporter | 0.13 | 2.71 | 0.28 | K |
| *emrE* | AWC34_RS11510 | QacE family quaternary ammonium compound efflux SMR transporter | -0.84 | 2.70 | -0.81 | EG |
| *lmrB^a^* | AWC34_RS00500 | MFS transporter | 0.44 | 2.53 | -1.01 | P |
| *lmrB^b^* | AWC34_RS12700 | MFS transporter | 0.32 | 2.86 | -0.90 | S |
| *mdtG* | AWC34_RS12295 | MFS transporter | 3.00 | 3.59 | 0.71 | S |
| *proP* | AWC34_RS11470 | MFS transporter | 2.83 | 2.07 | 1.69 | R |
| *secY* | AWC34_RS09225 | Preprotein translocase subunit SecY | 2.37 | 3.6 | 0.35 | P |
| *ydhP* | AWC34_RS02580 | MFS transporter | -0.65 | 3.66 | 0.52 | S |
| *ykkC* | AWC34_RS09590 | QacE family quaternary ammonium compound efflux SMR transporter | -0.49 | 2.12 | 0.64 | K |
| *sugE* | AWC34_RS00695 | QacE family quaternary ammonium compound efflux SMR transporter | -4.10 | -2.18 | -1.35 | P |
| *yfcA* | AWC34_RS01890 | Sulfite exporter TauE/SafE family protein | -3.39 | -0.62 | -0.40 | S |
| **Transporter** | |  |  |  |  |  |
| *benK* | AWC34_RS11310 | MFS transporter | -2.61 | -0.29 | -0.43 | P |
| *celA* | AWC34_RS00640 | PTS sugar transporter subunit IIB | -1.16 | -2.47 | -0.45 | G |
| *celC* | AWC34_RS00650 | PTS lactose/cellobiose transporter subunit IIA | -2.63 | -3.3 | -0.58 | G |
| *corA* | AWC34_RS09835 | Magnesium and cobalt transport protein CorA | -2.39 | -3.49 | -0.52 | G |
| *glpF* | AWC34_RS05240 | Aquaporin family protein | -1.32 | -2.3 | -0.35 | L |
| *hmuV* | AWC34_RS05805 | ABC transporter ATP-binding protein | -1.62 | -2.42 | -1.39 | R |
| *narT* | AWC34_RS10075 | NarK/NasA family nitrate transporter | -3.84 | -2.31 | -0.58 | S |
| *norB* | AWC34_RS10900 | MFS transporter | -2.23 | -0.71 | 0.13 | I |
| *rbsA* | AWC34_RS10985 | Bacteriocin ABC transporter ATP-binding protein | -2.91 | -3.38 | 0.03 | P |
| *tctB* | AWC34_RS11270 | Tripartite tricarboxylate transporter TctB family protein | -2.14 | -3.07 | -2.12 | E |
| *yadH* | AWC34_RS10210 | ABC transporter permease | -1.20 | -3.91 | -3.95 | S |
| *yiaM* | AWC34_RS12165 | TRAP transporter small permease | -2.11 | -2.46 | 0.25 | L |
| *yocR* | AWC34_RS00935 | Sodium-dependent transporter | -1.66 | -0.69 | -2.31 | E |
| *znuC* | AWC34_RS10205 | Lantibiotic ABC transporter ATP-binding protein | -1.11 | -2.02 | -1.56 | S |
| *ecfT* | AWC34_RS09175 | Energy-coupling factor transporter transmembrane protein EcfT | -0.18 | 2.37 | 0.74 | S |
| *sulP* | AWC34_RS11005 | SulP family inorganic anion transporter | -0.44 | 2.00 | 0.45 | S |
| **Salt tolerance** | |  |  |  |  |  |
| *betA* | AWC34_RS11515 | Choline dehydrogenase | -0.37 | 4.22 | 0.42 | V |
| *betB* | AWC34_RS11520 | Betaine-aldehyde dehydrogenase\|Betaine aldehyde dehydrogenase | 0.42 | 4.32 | 1.13 | ss |
| *ctrA* | AWC34_RS00080 | Amino acid permease | 0.18 | 3.10 | -1.05 | E |
| *mmuP* | AWC34_RS01740 | Amino acid permease | -0.19 | 2.13 | -0.75 | E |
| *nhaC* | AWC34_RS09730 | Sodium:proton antiporter | 0.00 | 3.00 | -0.45 | ss |
| *opuBD^a^* | AWC34_RS10315 | Choline transport system permease protein OpuBD | 0.41 | 2.07 | -1.72 | S |
| *opuBD^b^* | AWC34_RS10325 | Choline transport system permease protein OpuBD | 0.94 | 2.51 | -1.30 | S |
| *opuC^a^* | AWC34_RS07515 | Osmoprotectant ABC transporter substrate-binding protein | 1.84 | 3.75 | 0.99 | E |
| *opuC^b^* | AWC34_RS10320 | Osmoprotectant ABC transporter substrate-binding protein | 0.76 | 2.65 | -0.53 | S |
| *opuD^a^* | AWC34_RS05465 | Glycine betaine transporter OpuD | 1.71 | 3.69 | 0.38 | ss |
| *opuD^b^* | AWC34_RS09135 | Glycine betaine transporter OpuD | 1.87 | -0.07 | 2.62 | P |
| *opuD^c^* | AWC34_RS10865 | Glycine betaine transporter BetL | 1.76 | 4.16 | 0.33 | K |
| *pitA* | AWC34_RS02525 | Inorganic phosphate transporter | 0.24 | 2.22 | -0.86 | P |
| *putP* | AWC34_RS10580 | Osmoregulated proline transporter | 3.12 | 2.47 | 0.06 | EG |
| *treR* | AWC34_RS01010 | Trehalose operon repressor | 0.65 | 2.18 | 0.12 | G |
| *yeaV^a^* | AWC34_RS05770 | Glycine betaine transporter BetL | 0.56 | 3.82 | -0.71 | S |
| *yeaV^b^* | AWC34_RS11530 | L-carnitine/gamma-butyrobetaine antiporter | 0.78 | 4.83 | 0.23 | S |

**Supplementary Table 9.** Expression of two component system under chloramphenicol, erythromycin and lincomycin.

| **Gene locus** | **Product** | **COG** | **Log2(Fold change)** | | |
| --- | --- | --- | --- | --- | --- |
|  |  |  | **CHL** | **ERY** | **LIN** |
| AWC34_RS05855 | Sensor histidine kinase\|Signal transduction histidine-protein kinase ArlS | K | 0.66 | -0.35 | -0.24 |
| AWC34_RS05860 | DNA-binding response regulator\|Response regulator ArlR | K | -0.58 | -0.66 | 0.8 |
| AWC34_RS07115 | Sensor histidine kinase\|Sensor protein CiaH | L | 0.4 | 0.49 | -0.17 |
| AWC34_RS07120 | DNA-binding response regulator\|Alkaline phosphatase synthesis transcriptional regulatory protein PhoP | K | -0.13 | 0.32 | -0.41 |
| AWC34_RS07780 | DNA-binding response regulator\|Oxygen regulatory protein NreC | L | -0.08 | -0.44 | 0.89 |
| AWC34_RS07785 | Sensor histidine kinase\|Sensor histidine kinase YhcY | S | -0.01 | -0.27 | 0.32 |
| AWC34_RS08085 | DNA-binding response regulator\|Response regulator protein VraR | M | 0.11 | 0.22 | -0.07 |
| AWC34_RS08090 | Sensor histidine kinase\|Sensor protein VraS | R | -0.64 | -0.56 | 0.07 |
| AWC34_RS09905 | DNA-binding response regulator\|Transcriptional regulatory protein AfsQ1 |  | -0.09 | 0.6 | -0.23 |
| AWC34_RS09910 | Sensor histidine kinase\|Heme sensor protein HssS | P | 0.16 | 0.64 | -0.42 |
| AWC34_RS10080 | DNA-binding response regulator\|Oxygen regulatory protein NreC | K | -2.64 | -1.03 | -1.11 |
| AWC34_RS10085 | Sensor histidine kinase\|Oxygen sensor histidine kinase NreB | P | -2.4 | -1.42 | -0.96 |
| AWC34_RS12205 | DNA-binding response regulator\|Transcriptional regulatory protein ResD | S | -1.23 | 0.16 | 0.51 |
| AWC34_RS12210 | Sensor histidine kinase\|Signal transduction histidine-protein kinase ArlS | IQ | -0.27 | -0.18 | 0.9 |
| AWC34_RS12515 | Two-component system response regulator DcuR\|Probable C4-dicarboxylate response regulator DctR | C | 0.63 | -0.26 | -0.76 |
| AWC34_RS12520 | Two-component system sensor histidine kinase DcuS\|Probable C4-dicarboxylate sensor kinase | Q | 0.22 | -0.66 | -0.23 |
| AWC34_RS12690 | Cell wall metabolism sensor histidine kinase WalK\|Sensor protein kinase WalK |  | 1.13 | 0.68 | 0.34 |
| AWC34_RS12695 | DNA-binding response regulator\|Transcriptional regulatory protein WalR | L | 1.03 | 0.25 | 0.32 |


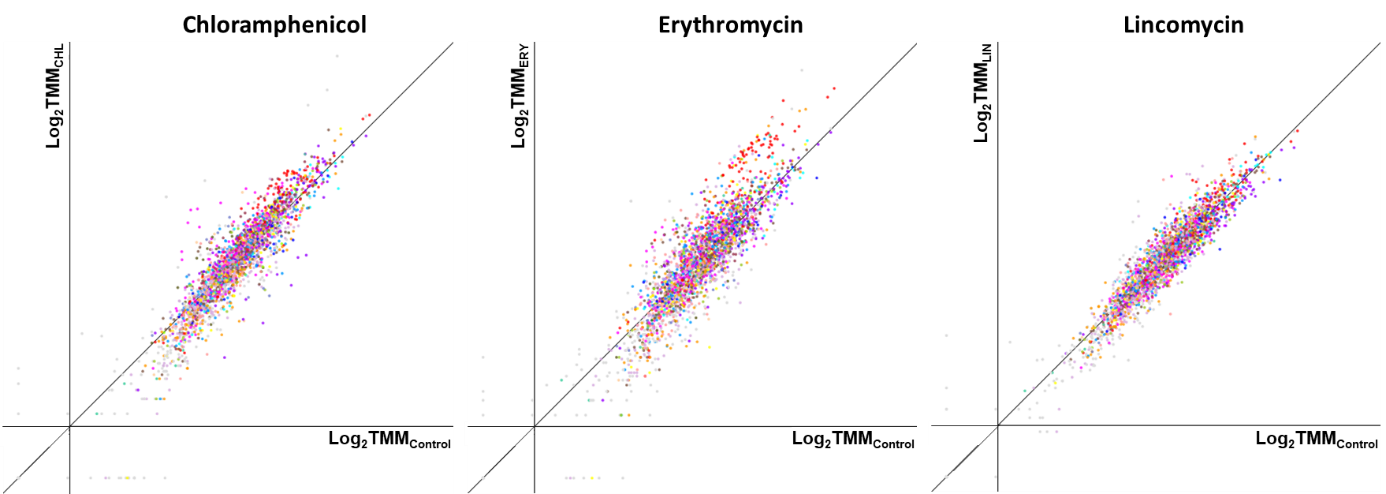


**Supplementary Figure 1.** DEG analysis in RNA-seq data between strain KM1031 and KM1031 with antibiotics. The x-axis shows the log-scaled TMM values of strain KM1031, and the y-axis shows the log-scaled TMM values with chloramphenicol, erythromycin, and lincomycin.


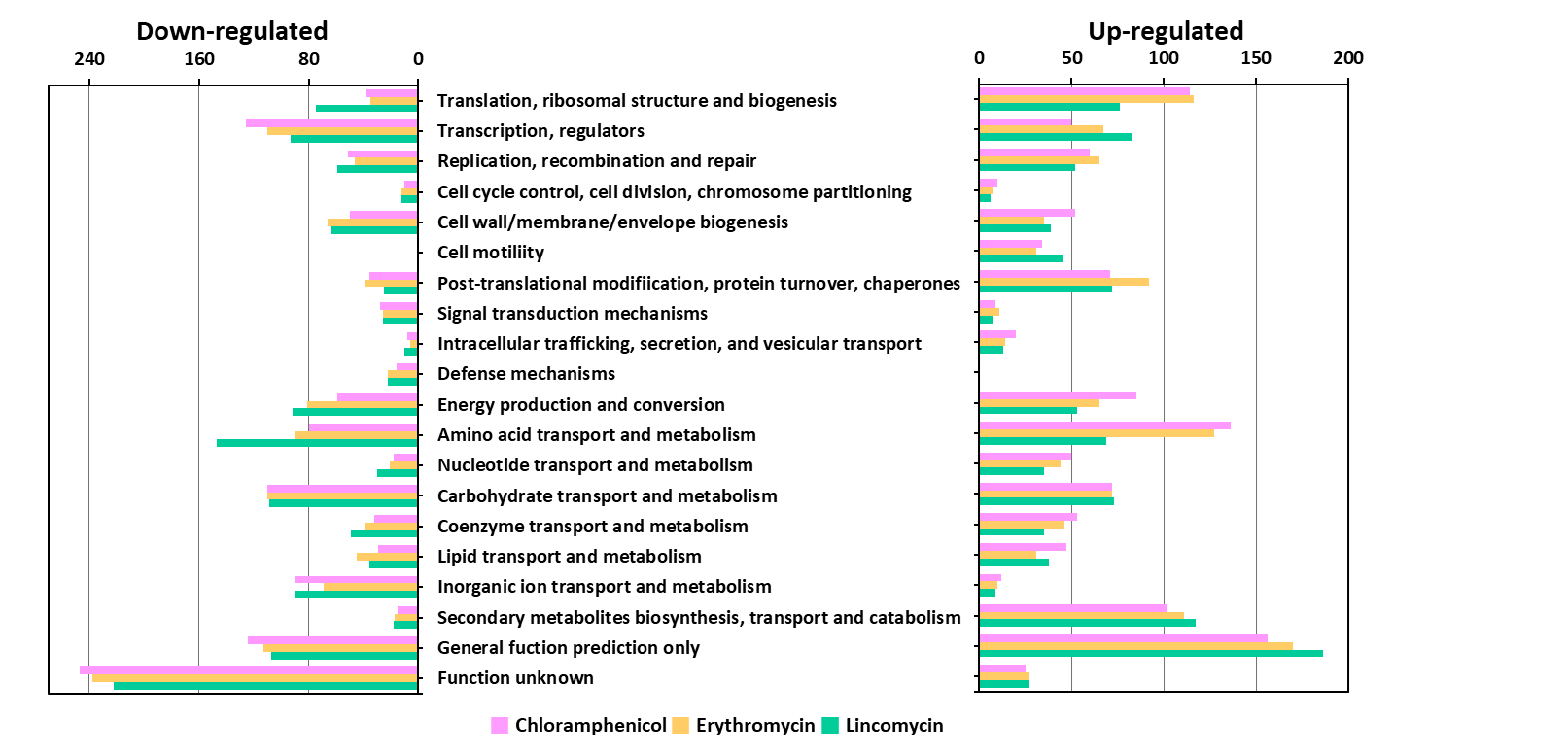


**Supplementary Figure 2.** Comparison of functional categories of strain KM1031’s RNAs under different antibiotics based on COG.


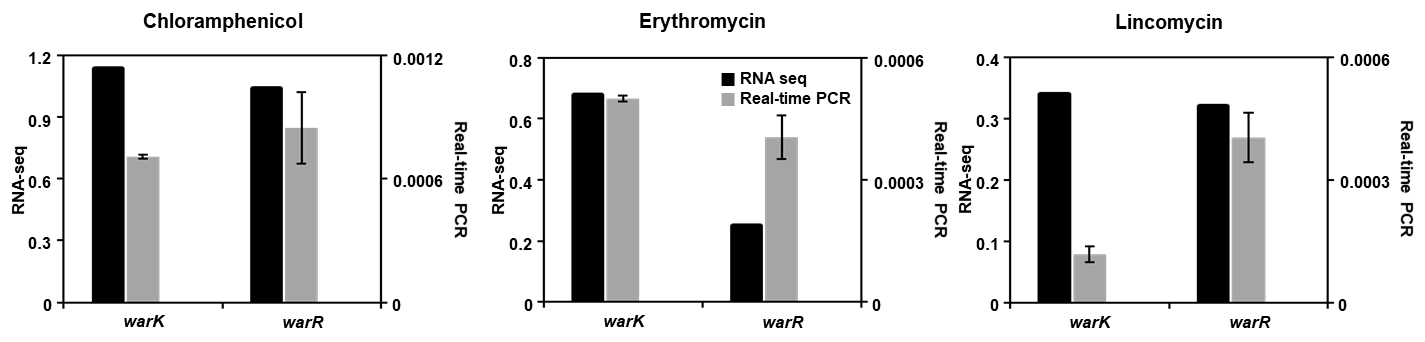


**Supplementary Figure 3.** RNA sequencing data and quantitative real-time PCR of *walKR* gene. RNA sequencing data expressed as log_2_fold-change in gene expression between control and antibiotic-treated samples. 16S rRNA gene expression was used as a control and transcripts of target gene were compared by real-time qRT-PCR.
